# A low-cost and open-hardware portable 3-electrode sleep monitoring device

**DOI:** 10.1016/j.ohx.2024.e00553

**Published:** 2024-07-06

**Authors:** Matías Rodolfo Pretel, Vanessa Vidal, Dante Kienigiel, Cecilia Forcato, Rodrigo Ramele

**Affiliations:** a Laboratorio de Sueño y Memoria, Life Sciences Department, Instituto Tecnológico de Buenos Aires (ITBA), Buenos Aires, Argentina; b Consejo Nacional de Investigaciones Científicas y Tecnológicas (CONICET), Buenos Aires, Argentina; c Computer Engineering Department, Instituto Tecnológico de Buenos Aires (ITBA), Buenos Aires, Argentina

**Keywords:** Open hardware, EEG, Arduino, Sleep, Portable device, Low cost

## Abstract

To continue sleep research activities during the lockdown resulting from the COVID-19 pandemic, experiments that were previously conducted in laboratories were shifted to the homes of volunteers. Furthermore, for extensive data collection, it is necessary to use a large number of portable devices. Hence, to achieve these objectives, we developed a low-cost and open-source portable monitor (PM) device capable of acquiring electroencephalographic (EEG) signals using the popular ESP32 microcontroller. The device operates based on instrumentation amplifiers. It also has a connectivity microcontroller with Wi-Fi and Bluetooth that can be used to stream EEG signals. This portable single-channel 3-electrode EEG device allowed us to record short naps and score different sleep stages, such as wakefulness, non rapid eye movement sleep (NREM), stage 1 (S1), stage 2 (S2), stage 3 (S3) and stage 4 (S4). We validated the device by comparing the obtained signals to those generated by a research-grade counterpart. The results showed a high level of accurate similarity between both devices, demonstrating the feasibility of using this approach for extensive and low-cost data collection of EEG sleep recordings.

## Graphical abstract

**Figure:**
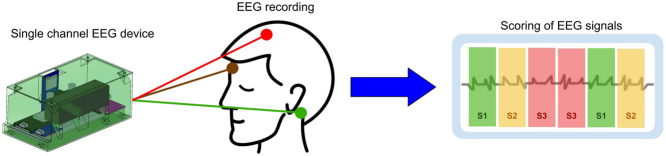

## Specifications table

**Table:** 

Hardware name	*Baby Blue*
Subject area	*Neuroscience*
Hardware type	*Physiological measurement*
Closest commercial analog	*InteraXon Muse 2*
Open source license	*GNU General Public License (GPL) v3.0**https://www.gnu.org/licenses/gpl-3.0.en.html*
Cost of hardware	<*U$D 100*
Source file repository	*Open Science Framework**https://doi.org/10.17605/OSF.IO/RJCHP*

## 1 Hardware in context

During the lockdown, as a result of the COVID-19 pandemic, experiments that were previously conducted in laboratories were shifted to the homes of volunteers. After several months of isolation, researchers learned about the importance of working with versatile tools that allowed them to carry out their experiments. In order to collect a large amount of sleep data, and since researchers did not have access to their laboratories, they had to use portable devices. The most affected were low-income countries, such as Latin American ones, because electroencephalography (EEG) portable devices are usually imported and highly expensive. Thus, the demand for low-cost and user-friendly portable devices arose. We aimed to develop a low-cost and open hardware EEG portable device to allow researchers from low-income countries to continue with their scientific research in the challenging pandemic context. We developed a 3-electrode portable EEG device called Baby Blue. This device is aiming specifically to conduct simultaneous out-of-the-lab sleep monitoring experiments with a limited budget. Hence, the design objectives of the device were low cost, portability, ease of use, and ease of set up. To the best of our knowledge, there is not any other open source device for this application.

A polysomnography (PSG) device is capable of monitoring various parameters and physiological signals during sleep [1]. It employs four to six EEG electrodes, two electrooculogram (EOG) electrodes and two electromyogram (EMG) electrodes. Sleep scoring is performed using these signals. Considering this, employing just a single EEG signal for this purpose poses a considerable challenge. However, multiple studies showcase the feasibility of conducting sleep scoring using a single EEG channel [2].

Sleep scoring based on standard criteria [3] consists of four stages of Non-Rapid Eye Movement (NREM) sleep, namely stage 1 (S1), stage 2 (S2), stage 3 (S3), and stage 4 (S4), followed by Rapid Eye Movement (REM) sleep. Slow-Wave Sleep (SWS) encompasses the deepest stages of NREM sleep, specifically S3 and S4. Each stage is characterized by distinct electroencephalographic (EEG) signal features and associated physiological states. S1 is the initial stage of sleep, marking the transition from wakefulness. This stage is characterized by low-amplitude, mixed-frequency EEG signals, with a reduction in alpha activity (8–13 Hz) and an increase in theta activity (4–8 Hz). Physiologically, there is a decrease in muscle activity, the presence of slow eye movements, and slight reductions in heart rate and respiration. S2 follows, identified by the appearance of sleep spindles (bursts of 12–15 Hz activity lasting about 0.5–2 s) and K-complexes (sudden, high-amplitude waves). In this stage, muscle activity continues to decline, eye movements cease, and both heart rate and body temperature decrease, signifying a deeper stage of sleep. S3 marks the onset of deep sleep, characterized by high-amplitude, low-frequency slow oscillations (0.5–1 Hz) and delta waves (1–4 Hz) in the EEG. Physiologically, muscle tone is significantly reduced, and it becomes very difficult to awaken the sleeper. Heart rate and respiration are at their lowest, highlighting the stage’s importance for physical restoration and recovery. S4, the deepest stage of NREM sleep, is dominated by slow wave activity (0.5–4 Hz) in the EEG, indicating profound sleep depth. These oscillations play a fundamental role in memory consolidation and synaptic plasticity, facilitating the transfer of information from short-term to long-term memory storage [4]. During this stage, the body reaches its most relaxed state, with minimal muscle activity, extremely slow heart rate, and respiration.

Baby Blue enables the recording of EEG signals from short naps, with manual scoring of different stages of sleep after the signal acquisition by two experts according to standard criteria (as wakefulness, NREM, S1, S2, S3 and S4) [3]. This device does not replace other higher complexity devices; however, it guarantees the minimum requirements to achieve the objectives of sleep research studies at a much lower cost. For example, BrainAmp DC (Brain Products GmbH, Munich, Germany) devices can measure biological signals with high signal quality, but are intended for use within a laboratory and are often very expensive (U$D 30.000 approx). There are many development boards that are less expensive and allow us to acquire up to 16 bio-signals simultaneously, for example, the OpenBCI Cyton board [5] (greater than U$D1000), but it is a very high cost when many devices are required. In this study, we describe the design of a very low-cost device (less than U$D 100), which is portable, has great autonomy, and uses open source technology.

We conducted a comparative analysis of Baby Blue’s technical specifications alongside its market counterparts. Our findings suggest that Baby Blue meets the required objectives and we highlight its limitations and potential areas for enhancement. Furthermore, it demonstrates its utility in situations demanding extensive field studies beyond the confines of a traditional laboratory environment.

### 1.1 Comparison between devices

Since our device was designed for parallel simultaneous concurrent studies, we have taken into account the devices available on the market that meets similar characteristics. A device comparable to ours should have the following characteristics. Firstly, it should be priced at less than U$D 500 to ensure accessibility. Secondly, portability is essential, enabling seamless transportability beyond the confines of the laboratory environment. Lastly, an efficient design with minimal electrodes is crucial to ensure ease of use and user-friendliness.

When comparing Baby Blue with several similar devices [6] (Table 1), we observe that Baby Blue has notable features such as battery capacity, data transmission capability, customization options for specific requirements, and a significantly lower cost compared to other devices.

**Table 1:** Comparison between portable EEG devices with Baby Blue.

Device	Baby Blue	MUSE 2	MUSE S	NeXus-10 MKII	Ganglion
Company	–	InteraXon	InteraXon	Mind Media	OpenBCI
Year market release	–	2018	2021	–	2016
Number of EEG electrode	3	4	4	4	4
Electrode placement	Flexible	Fixed	Fixed	Flexible	Flexible
Reference location	Flex	FPz	FPz	E/M	E
Accelerometer	No	Yes	Yes	–	Yes
Gyroscope	No	Yes	Yes	–	No
Bipolar ExG	0	0	0	4	–
Aux	0	2	2	4	–
Triggers	No	No	No	Yes	–
Electrode type	Wet, Gel	Dry	Dry	Gel	Dry. Wet, Gel
Electrode preamplification	Passive	Passive	Passive	Passive	Passive
Electrode shielding	Shielded	Shielded	Shielded	Shielded	Shielded
Impedance check	No	No	No	–	Start/Checked
Input range (mVpp)	0.33	2	2	200	3000
Input impedance (GΩ)	10	–	–	1	0.1
Input noise (μVrms)	3	–	–	1	0.05
CMRR (db)	80	–	–	90	106
Bandwidth (Hz)	40	100	100	–	150
Sampling rate (Hz)	256	256	256	2048	200
Resolution (bits)	12	12	12	24	24
Least significant bit (μV/bit)	0.08	0.488	0.488	0.012	0.179
Size (mm)	135 × 90 × 60	–	–	140 × 120 × 45	61 × 61 × 30
Weight (g)	80	39	41	540	30
Battery (h)	24	5	10	24	8
Wireless protocol	Wi-Fi/BLE	Bluetooth	Bluetooth	Bluetooth	BLE
Local storage/SD card	SD	No	No	SD	SD
Output format	txt	CSV. edf	CSV, edf	txt, mat, edf	txt, bdf
Smartphone EEG	No	Yes	Yes	No	Yes
Medical certification	No	No	No	Yes	No
Price range (U$D)	<100	<500	<500	–	<1000

## 2 Hardware description

**Fig. 1:**
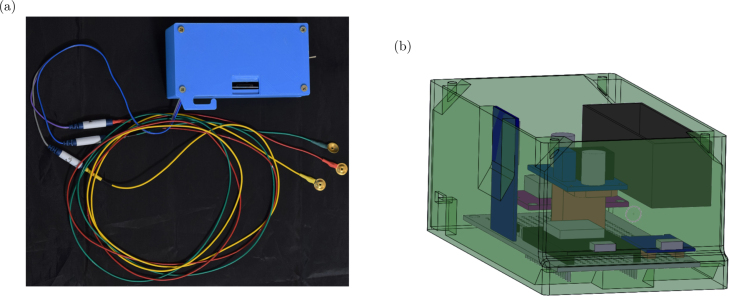
(a) Image of Baby Blue with its three electrodes. (b) 3D model of Baby Blue designed with SolidWorks.

Baby Blue (Fig. 1(a)) is based on the ESP32 [7] microcontroller programmed with the Arduino (Arduino LLC, Italy) development environment. It simplifies the acquisition of EEG data using only three electrodes via a microcontroller. The microcontroller captures EEG signals from an operational amplifier integrated into the SparkFun Single Lead Heart Rate Monitor AD8232 (SparkFun LLC, Niwot, Colorado), which was originally designed for electrocardiogram (ECG) signals [8], [9]. We have made essential modifications to adapt it for EEG signal recording. Once the digital signal is obtained, the data is stored on a removable micro SD memory card, enabling post-processing by researchers. Additionally, the device features a rechargeable battery, providing long autonomy for use during naps or throughout the night. This makes it a portable and cost-effective solution suitable for various studies with extensive data collection requirements. Compared to other EEG devices such as OpenBCI’s Cyton Board, Baby Blue stands out for its user-friendliness, as it does not require a graphical interface for its operation. However, Baby Blue can transmit data via both WiFi and Bluetooth, providing the flexibility to enhance the design based on user preferences and needs.

Baby Blue is powered by a rechargeable 3.7 V battery (model: 18650) with a capacity of 3000 mAh, providing approximately 30 h of autonomy. The integrated AD8232 consumes 27.5 mA, the micro SD memory consumes 25 mA, and the ESP32 microcontroller consumes 20 mA (in modem sleep mode, with WiFi, Bluetooth, and radio inactive). The battery is connected to a Li-ion battery charging and discharging module (M1) featuring the TP4056A integrated circuit. The TP4056 is a comprehensive constant-current/constant-voltage linear charger designed for single-cell lithium-ion batteries. It maintains a fixed charge voltage of 4.2 V and a programmable charge current of 1000 mA. Thus, it is recommended to use a USB charger providing 5 V and a current greater than 1 A. Furthermore, the module includes a protection circuit offering several features: Over-discharge protection ensures the battery is not discharged below 2.4 V, a healthy minimum voltage level. If the battery voltage drops below 2.4 V, the module will cut off output power until the voltage reaches 3.0 V (the over-discharge release voltage), at which point it will resume power delivery to the load. Overcharge protection ensures safe charging to 4.2 V. Overcurrent and short-circuit protection will disconnect the battery output if the discharge rate exceeds 3 A or in the event of a short circuit. Soft-start protection limits inrush current. The output of M1 is connected to a boost-type switching power supply module (M2) via a single-pole double-throw switch that allows cutting off the current supply to the rest of the circuit. M2 elevates the battery voltage and regulates it to 5 V at its output. This regulated 5 V voltage is then applied to the power input of the NodeMCU ESP32 module (M3), which features a linear regulator providing a 3.3 V supply for both the microcontroller, AD8332, and the micro SD memory.

The ESP32 microcontroller integrates two 12-bit analog-to-digital converters (ADCs) utilizing successive approximation register (SAR) methodology for accurate conversion. With the Wi-Fi module enabled, its sampling rate can achieve up to 1000 samples per second, while theoretically supporting a maximum sampling rate of 2 MHz. The ADC input range should be maintained within 0 V to 3.3 V, with a Least Significant Bit (LSB) resolution of 0.805 μV.

Key features of Baby Blue include:

• Cost-efficiency and ease of use, making it accessible to individuals with minimal expertise.
• Versatility and ease of programming, providing the potential to utilize its Wi-Fi and Bluetooth transceiver features for additional improvements.
• Suitability for sleep studies and other EEG experiments that span multiple hours.

## *Design files*

**Fig. 2:**
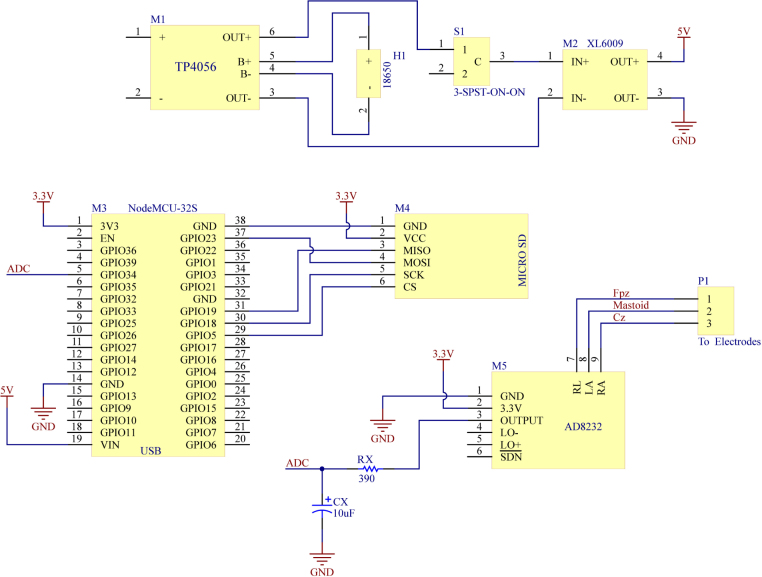
Electronic layout of the Baby Blue.

The enclosure was designed with SolidWorks (Dassault Systemes Solidworks Corporation, USA), and the 3D models were printed using polylactic acid (PLA) plastic filament with an Artillery Genius Pro 3D printer (Fig. 1(b)). The electronic layout is detailed in Fig. 2, and the firmware was developed using the Arduino IDE (version 1.8.19). All design files, including the assembly instructions, can be found in our Open Science Framework repository ( https://doi.org/10.17605/OSF.IO/RJCHP).

## 3 Design files summary

This section provides an overview of the files associated with the project. It includes files containing 3D models for physical components such as the case and various component holders (STL files), schematics for electronic circuits (PDF file), source code for microcontroller firmware (INO file), and a Bill of Materials (BOM) detailing the necessary components for construction (XLSX file) (see Table 2, Table 3).

**Table 2:** **Battery charger holder.STL**: File providing the 3D model to print the battery charger holder. **Main case.STL**: File providing the 3D model to print the enclosure. **Switching regulator holder.STL**: File providing the 3D model to print the switching regulator holder. **Top cover.STL**: File providing the 3D model to print the cover of the enclosure. **EEGOne PCB Schematic.PDF**: File providing the electrical circuit of the device. **ESP32_firmware.ino**: Arduino project containing the source code. **BOM.xlsx**: bill of materials file.

Design filename	File type	Open source license	Location of the file
Battery charger holder.STL	STL	GNU General Public License (GPL) v3.0	https://doi.org/10.17605/OSF.IO/RJCHP
Main case.STL	STL	GNU General Public License (GPL) v3.0	https://doi.org/10.17605/OSF.IO/RJCHP
Switching regulator holder.STL	STL	GNU General Public License (GPL) v3.0	https://doi.org/10.17605/OSF.IO/RJCHP
Top cover.STL	STL	GNU General Public License (GPL) v3.0	https://doi.org/10.17605/OSF.IO/RJCHP
EEGOne PCB Schematic.PDF	CAD	GNU General Public License (GPL) v3.0	https://doi.org/10.17605/OSF.IO/RJCHP
ESP32_firmware.ino	INO	GNU General Public License (GPL) v3.0	https://doi.org/10.17605/OSF.IO/RJCHP
BOM.xlsx	XLSX	GNU General Public License (GPL) v3.0	https://doi.org/10.17605/OSF.IO/RJCHP

## 4 Bill of materials summary

The following bill of material shows the components necessary to build the device presented in this project. It should be noted that if there is an intention to modify the device for use in other applications, it is possible that the values of the passive components may need to be altered, such as those in the anti-alias filter. For those seeking a deeper understanding of the components used in this project, we recommend consulting the materials list available in the repository ( https://doi.org/10.17605/OSF.IO/RJCHP). This resource provides comprehensive information on each component, including detailed descriptions, part numbers, and relevant supplier links.

**Table 3:** Bill of materials: The * symbol refers to the component tags C1, C3, and R8 that correspond to the components delineated in the schematic circuit provided by Sparkfun [10]. The # symbol refers to a touch proof type electrode adapter marketed by OpenBCI. This adapter will depend on the electrode being used.

Designator	Component	Number	Cost per unit	Total cost	Source of materials	Material type	Tolerance
H1	Battery holder	1	$USD 4.54	$USD 4.54	Digikey	Non-specific	
	18560 Li-ion battery	1	$USD 6.95	$USD 6.95	Digikey	Lithium	
M3	ESP32	1	$USD 10	$USD 10	Digikey	Non-specific	
	Perfboard	1	$USD 4.29	$USD 4.29	Digikey	FR4 Epoxy Glass	
S1	Switch SPDT (ON-ON)	1	$USD 0.99	$USD 0.99	Digikey	Non-specific	
M2	Switching regulator step-up	1	$USD 7.50	$USD 7.50	Digikey	Non-specific	
M5	Heart rate monitor module	1	$USD 21.50	$USD 21.50	Digikey	Non-specific	
M4	Micro SD shield module	1	$USD 4.49	$USD 4.49	Amazon	Non-specific	
	Micro SD memory card	1	$USD 12	$USD 12	Digikey	Non-specific	
M1	Battery charger	1	$USD 1.65	$USD 1.65	Addicore	Non-specific	
C1*	Capacitor 330 pF	1	$USD 0.16	$USD 0.16	Digikey	Ceramic	1%
C3*	Capacitor 27 nF	1	$USD 0.11	$USD 0.11	Digikey	Ceramic	1%
R8*	Resistor 100 k℧	1	$USD 0.10	$USD 0.10	Digikey	Thick film	1%
RX	Resistor 390 ℧	1	$USD 0.10	$USD 0.10	Digikey	Carbon film	1%
CX	Capacitor 10 μF	1	$USD 0.26	$USD 0.26	Digikey	Aluminum electrolytic	20%
EA#	Electrode adapter	1	$USD 29.99	$USD 29.99	OpenBCI	Non-specific	

## 5 Build instructions

### 5.1 Component modification

**Fig. 3:**
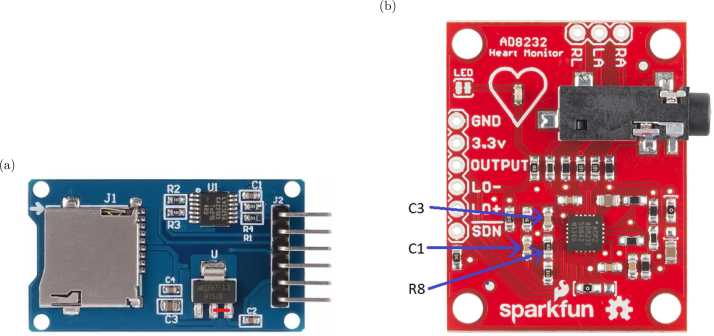
(a) Micro SD card module. The short circuit to be created is marked in red. (b) Heart rate monitor module based on AD8232 implemented on a SoC from the Sparkfun (SparkFun LLC, Niwot, Colorado) provider. The image shows the electronic components that need to be replaced.

**Fig. 4:**
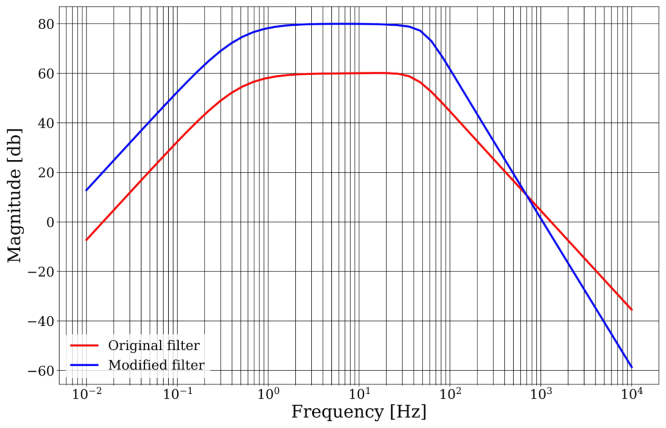
Comparing Frequency Responses: Original vs. Modified Filters. The image shows the frequency response of the original filter implemented in the heart rate monitor module (red line), and the frequency response of the modified filter (blue line). Both curves were obtained using the *AD8232 Filter Design Tool*[15] and plotted with the *bode* function of the *control* library (version 0.9.3).

Before making the electrical connections between different modules and components, some modifications are necessary:

• Adaptation of the working voltage of the microSD memory reader The SD memory card module is designed to operate at 5 V, while the overall system of the device operates at 3.3 V. For this reason, the module needs to be adapted to accept 3.3 V instead of 5 V. The module contains a linear voltage regulator that converts 5 V to 3.3 V. To bypass the voltage regulator, it is necessary to short-circuit the input with the output, as shown in Fig. 3(a). This procedure requires the use of solder and a soldering iron.
• ECG Module Modification The Heart Monitor module AD8232, illustrated in Fig. 3(b), contains a differential amplifier with a total gain of 60 dB, suitable for amplifying ECG signals. However, this gain is insufficient to amplify EEG signals, which are in the microvolt range [11], [12]. Therefore, it became necessary to modify the circuit within the module, increasing the gain to approximately 80 dB. To carry out this modification, three components need to be replaced: capacitor C3 (10 nF) with a 27 nF capacitor, C1 (1.5 nF) with a 330 pF capacitor, and resistor R8 (100 kΩ) with a 10 kΩ resistor. The heart rate monitor module has a second-order Sallen Key bandpass [13] filter, with passband frequencies of 0.48 Hz and 41.09 Hz, providing a passband gain of 60 dB. With these modifications, the passband cutoff frequencies have been adjusted to 0.48 Hz and 53.32 Hz, and the passband gain has been increased to 80 dB, which is enough for amplifying microvolt-level signals. The specifications of Baby Blue are finely tuned for utilization in sleep studies, targeting the specific frequency bands of Delta, Theta, Alpha, and Beta that are of primary interest. In our investigation, the exclusion of the Gamma frequency band (>30 Hz) was deliberate, along with precautions taken to minimize potential 50 Hz noise interference. That is why an external low-pass RC filter with a cutoff frequency of 40.81 Hz has been incorporated (see Fig. 2). In case the low gamma (30–45 Hz) and fast gamma (45–60 Hz) frequency bands [14] are of interest, a replacement of CX with a 3.3 μF capacitor and C3 with an 18 nF capacitor could be conducted. With these values, the cutoff frequency of the low-pass filter will be 65.3 Hz, and the anti-aliasing filter will have a cutoff frequency of 123 Hz. It is important to note that the signal may exhibit a higher presence of the 50 Hz component. However, the flexibility of Baby Blue’s design parameters allows for customization to include the Gamma frequency band if necessary, offering researchers adaptability in their studies. We present a graphical representation comparing the frequency response curves of two filters: the original filter and its modified counterpart (see Fig. 4). It is important to note that the frequency response curves were obtained numerically through calculation methods rather than experimental measurements. It is suggested that experimental measurements be considered to validate the filter response.

### 5.2 Device assembly

**Fig. 5:**
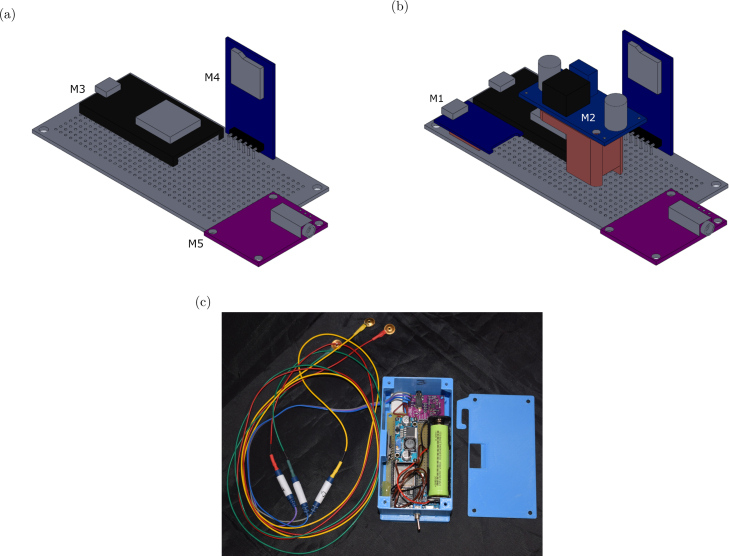
(a) Image showing the placement of the components (M3, M4 and M5) on the perfboard. (b) The perfboard including 3D printed holders for the battery charger (M1) and switching voltage regulator (M2). (c) Image showing the internal view of Baby Blue with its exposed electronic components.

• Mounting components on a perfboard Components M3, M4, M5, RX, and CX are soldered components. This means that these modules and components must be mounted on a 100 × 50 mm perfboard, as illustrated in Fig. 5(a), and the electrical connections indicated in the schematic diagram of Fig. 2 should be made. Components M1, M2, H1, and S1 are non-soldered components, which implies that they will be attached to the board using plastic adhesive and will not be directly soldered onto the board. Nevertheless, electrical connections need to be established using wires. To secure the battery charger (M1) and the switching regulator (M2), you can use the plastic holders (Fig. 5(b)) whose 3D models are available in our repository (*Battery charger holder.STL* and *Switching regulator holder.STL*).
• Mounting the main board into the enclosure Once all the electrical connections have been made, the board should be inserted along with its components inside the 3D-printed enclosure (Fig. 5(c)). To secure the board with its components, battery holder, and power switch within the enclosure, it is recommended to use plastic adhesive. Finally, the enclosure must be closed by fastening four self-tapping screws (e.g., 3.5 mm in diameter and 10 mm in length). After completing this step, the microSD memory card should be inserted.
• Firmware Upload It is important to have the Arduino IDE version 1.8.x installed, which can be downloaded from the official Arduino website. Additionally, it is required to download the *Esp32_Firmware.ino* project. For detailed instructions on how to load the firmware into the ESP32 microcontroller, refer to the documentation in our repository ( https://doi.org/10.17605/OSF.IO/RJCHP).

## 6 Operation instructions

Baby Blue operates with three electrodes (ground, reference, and signal). These electrodes are connected to the Heart Rate Monitor Module through a three-terminal connector labeled as follows: RA (right arm), LA (left arm), and RL (right leg), see Fig. 3(b). The signal electrode will be connected to the RA terminal, the reference electrode to the LA terminal, and the ground electrode to the RL terminal. According to the electrodes employed, the appropriate adapter should be used. In our specific setup, we employed OpenBCI’s Header pin to touch-proof electrode adapter to establish the connection with the heart rate monitor module. Prior to electrode placement, the area is prepared with alcohol and abrasive gel to reduce skin impedance. Both are applied using swabs, gently cleaning the skin. Subsequently, a small quantity of conductive cream is applied to the electrode, which is then placed on the prepared area, applying gentle pressure. Finally, hypoallergenic adhesive tape is used to secure the electrodes in position.

After placing the electrodes, Baby Blue is turned on using the on-off switch, and signal acquisition begins. A red light indicates that the equipment is operating correctly. However, if the SD memory is not working properly or is not inserted in the micro SD memory card reader module when the equipment is turned on, a blue light will start flashing.

To conclude signal acquisition, the on/off switch can be used to turn off the device. After removing the electrodes, the experimenter must clean the skin with alcohol to eliminate any remnants of abrasive gel and conductive cream.

The data stored in the SD memory will be saved into a file named “eeg.dat”. Each sleep record will be stored after the last one recorded. Every time the device is powered on, it will write “New recording” into the file, followed by the data in binary format (four bytes for the sample counter plus two bytes for the sample value). Incorporating a real-time clock into the design offers enhanced control over the records stored in the SD memory. This addition enables precise timestamping, facilitating better management and organization of data, thus optimizing overall system functionality. In our design, we chose not to incorporate it to minimize costs.

## 7 Validation and characterization

To validate the proposed device, we conducted a verification experiment where the operational performance was contrasted against a research grade EEG device.

### 7.1 Subjects

Ten participants volunteered for the study (M: 25 years, SD: ±4). They signed a written informed consent approved by the Human Ethics Committee of the Universidad de Buenos Aires (Buenos Aires, Argentina) prior to participation in the study.

### 7.2 Procedure

Participants arrived in the sleep lab after midday and were allowed to sleep for 40 min while a polysomnography was performed with two devices simultaneously, the Baby Blue device and a 32-channel BrainAmp DC amplifier (Brain Products GmbH, Munich, Germany).

#### 7.2.1 Equipment and electrodes position

Before electrode placement on the skin, the surface was cleaned and disinfected with ethyl alcohol. For the BrainAmp DC device, electrodes were located according to the 10–20 International System, in position Cz referenced to A1 (left mastoid) with a ground lead to Fpz. Additionally, two electrodes measured the EOG (located 1 cm left of the left eye canthus and 0.5 cm down, and 1 cm right of the right eye and 0.5 above right canthus), and EMG located 1 cm left and right of the chin. For Baby Blue, electrodes were placed approximately 1 cm backwards Cz, A1 and Fpz. Electrodes for both devices were located as close as possible between them without physically touching. Passive Ag/AgCl electrodes were used, with Tens conductive gel. The impedance for BrainAmp DC device was below 10 kΩ.

In the context of EEG, impedance is commonly assessed by applying a small alternating current between two or more electrodes. By measuring the voltage difference between the electrodes and knowing the applied current, the resistance can be determined. The current magnitude is typically chosen within the range of 5 nA to 25 μA, with a frequency selected between 10 and 40 Hz [16]. When measuring the impedance between two electrodes, the measured value reflects the impedance of everything between the two electrodes, including the impedance between each electrode and the live skin tissue directly beneath it (with a small contribution from the impedance of tissues between the two electrodes). However, it is possible to estimate the impedance between each individual electrode and the underlying live skin [17].

Although Baby Blue currently lacks the capability to measure electrode impedance, future work for this development will involve incorporating an extra module to measure impedance and utilizing sound alerts to maintain the low-cost requirement of the device. To address this limitation, we connected the electrodes previously positioned on the participant’s scalp to the BrainAmp DC. Using the BrainVision Recorder Software (Version 1.24.0101), we obtained impedance values. Once we confirmed that the impedance was sufficiently low (<10 kΩ) to ensure a high-quality signal acquisition, they were connected back to Baby Blue for signal acquisition.

It should be noted that the validation was performed with the particular electrode montage hitherto described, and the same impedance values may not be guaranteed with another configuration.

### 7.3 Data acquisition

Data acquisition was obtained for both devices at the same time, with a sampling frequency of 250 Hz. The single channel data obtained from the BrainAmp DC device was transmitted into a workstation computer. On the other hand, the data from the Baby Blue device was dumped into an SD card in binary format, using 6 bytes, where the first 4 bytes were used for circular counter, acting like a timestamp, plus 2 bytes to hold the 12 bits from the ADC conversion, obtaining a LSB resolution of 0.0798μV (with an amplifier gain of 80 dB), compared to BrainAmp DC which has an LSB of 0.5 μV (at a sampling rate of 250 Hz).

### 7.4 Data processing

Data processing was performed offline using Python version 3.9. All EEG data was processed with a Butterworth second-order bandpass filter from 0.16 to 35 Hz with a bandwidth of 34.84 Hz. Additionally, a 50 Hz notch filter with a quality factor of 30 and a bandwidth of 1.6 Hz was applied to remove interference (Q =ω0/bw). The coefficients for the bandpass filter were calculated using the *signal.butter* function, while the *signal.iirnotch* function was used for the notch filter coefficients. Subsequently, both filters were applied using the *signal.filtfilt* function. All of these functions are part of the *Scipy* library (version 1.10.1).

The cross-correlation illustrates the temporal relationship between two signals. The peak in the graph denotes instances of notable similarity between the signals, with the peak occurring at time zero indicating a moment of high correlation where the signals align without temporal displacement, signifying strong agreement between them [18]. We computed the autocorrelation of the signal obtained from BrainAmp DC, which represents the correlation of the signal with itself. Additionally, we computed the cross-correlation between the signals captured by both devices. Cross-correlation between signals was calculated to determine any time delay between them. After determining the delay time, it was subtracted from one signal to align both. This procedure allowed obtaining the time lag between signals and to synchronize them in time. To ensure consistency in the length of the records obtained by both devices, it was determined to extract the remaining samples from the record with the longest duration. These samples delineate the time period when the volunteer transitioned from sleep to wakefulness after the nap.

### 7.5 Data analyses and statistics

BrainAmp DC recordings were scored offline by two experts, identifying wakefulness and sleep stages 1–4. To ensure comparability with Baby Blue recordings, the scoring process considered data from the Cz scalp electrode, electromyographic, and electrooculographic recordings. Baby Blue recordings underwent the same scoring process, albeit without the EOG and EMG data.

The absence of these signals (EOG and EMG) makes it difficult to detect arousals and distinguish wakefulness from S1. A total of 857 epochs were obtained from each device across the 10 records, with each epoch representing a 30-s segment. After the scoring process was completed, confusion matrices were computed to assess the predictive accuracy for each sleep stage. Also, the inter-annotator agreement (Cohen’s kappa) was calculated using the list of stages among scorers, yielding a value of κ = 0.71.

To calculate the level of similarity of the signals measured by both devices, we calculated the intraclass correlations (ICC) [19], [20], [21], [22], [23], [24], [25]. For each record, 30-s segments labeled with the same stage by both scorers were extracted. These segments were then synchronized using cross-correlation and segmented into 2-s intervals. Finally, the ICC coefficient was calculated using the *pingouin.intraclass_corr* function from the *Pingouin* library (version 0.5.4).

We calculated individual average power spectra, using Brain Vision Analyzer (version 2.2, Brain Products). 30-s epochs were divided in 10-s segments artifact-free (BrainAmp DC recordings) or 10-s segments with artifacts (Baby Blue), allowing an overlap of 5 s in time. Each block was tapered by a single Hanning window before applying Fast Fourier Transformation that resulted in block power spectra with a frequency resolution of 0.061 Hz. Power spectra were then averaged across all blocks (Welch’s method). Mean power over central electrodes was determined for the frequency bands of interest, slow oscillations (0.5–1 Hz), delta (1–4 Hz), theta (4–8 Hz), slow spindle (9–12 Hz), fast spindle (12–15 Hz), beta (15–30 Hz), and alpha (8–13 Hz).

A Bland-Altman analysis was conducted to assess the concordance of power measurements across individual frequency bands and sleep stages. The Bland-Altman analysis is a method used to assess the agreement between two quantitative measurements or instruments. It involves plotting the differences between the two measurements against their averages. The resulting Bland-Altman plot provides insights into the level of agreement, any systematic bias, and the variability between the two methods. Plots were created using the *matplotlib* library (version 3.5.1).

### 7.6 Results and discussion

**Fig. 6:**
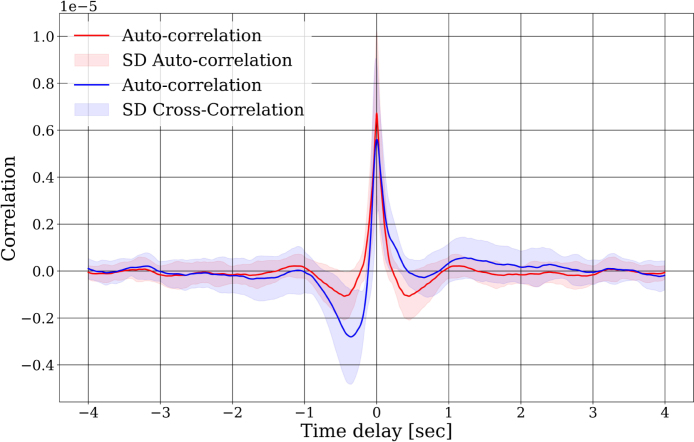
Cross correlation between the two signals (Bably Blue and BrainAmp DC). The blue curve shown in the figure represents the average cross-correlation between the signals acquired from both devices. The prominent peak at zero indicates a high degree of synchronization without any lag. Additionally, the shaded area around the average curve represents the standard deviation, providing insights into the variability of the correlation. The figure also includes the autocorrelation of the signal acquired with BrainAmp DC (red curve), enabling a comparative analysis with the cross-correlation.

We first analyzed the correlation between the EEG signals obtained from both devices and compared it with the autocorrelation obtained for the BrainAmp DC signals. We observed that the cross-correlation resembles an impulsive response. Furthermore, its similarity to autocorrelation is evident, and this indicates a high degree of similarity. The fact that both peaks are temporally aligned indicates that the devices are capturing synchronized information without any delay. Such findings suggest that Baby Blue ensures a consistent acquisition of EEG signals (Fig. 6). Also, we confirmed the similarity of the signals obtained, as indicated by high intraclass correlation values (ICC > 0.77) for all sleep stages (Fig. 9). Regarding the classification of sleep stages, of the 867 epochs, a total of 118 epochs were classified as wakefulness from both devices, 156 epochs as S1, 408 as S2, 110 as S3, and 75 as S4. The confusion matrices depicted in Fig. 8 highlight the percentages of classifications based on the sleep stage. For instance, in Fig. 8(a), of the 118 epochs classified as wakefulness, 114 (96.8%) were accurately identified using Baby Blue acquired data, while 5 (3.2%) were mistakenly labeled as S1. We have observed that S2 and S4 could be detected with high precision (Fig. 8(a)). Detecting the S3 stage was possible, even with a slightly lower accuracy compared to S2 and S4. This difference could be due to the different placement of the electrodes. While the electrode of the Brain Vision is located at Cz, the electrode used by Baby Blue is approximately 1 cm backwards. The amplitude of the signals may vary due to this separation, and it could influence the scoring of sleep stages. S1 was scored as wakefulness many times, however, the experts were able to correctly classify the wakefulness. To enhance the detection of the S1, the inclusion of an EOG channel is crucial because during S1 there are no rapid eye movements, but they are present during wakefulness. On the other hand, Baby Blue had a good performance in detecting SWS (Fig. 8(b)) and NREM stages (Fig. 8(c)).

**Fig. 7:**
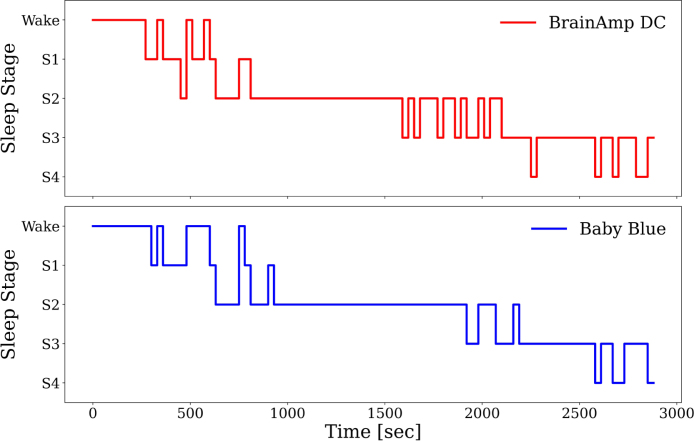
Hypnogram for the same subject obtained by analyzing independently signals from both devices.

Fig. 7 shows the hypnogram performed by an expert, corresponding to a volunteer who reached stage S4 of sleep. Additionally, it illustrates the similarities and differences between the hypnograms obtained using both devices. Although the general shape of the hypnograms is similar, we can observe that the expert encounters some difficulty in identifying transitions between stages. For example, between seconds 1500 and 2000, we observe transitions between stages that are unique to the hypnogram obtained with six channels. As mentioned before, this difference could be attributed to the different placement of the electrodes, which were approximately 1 cm apart. Consequently, the signals are not exactly the same, resulting in a difference in the classification of the stage.

**Fig. 8:**
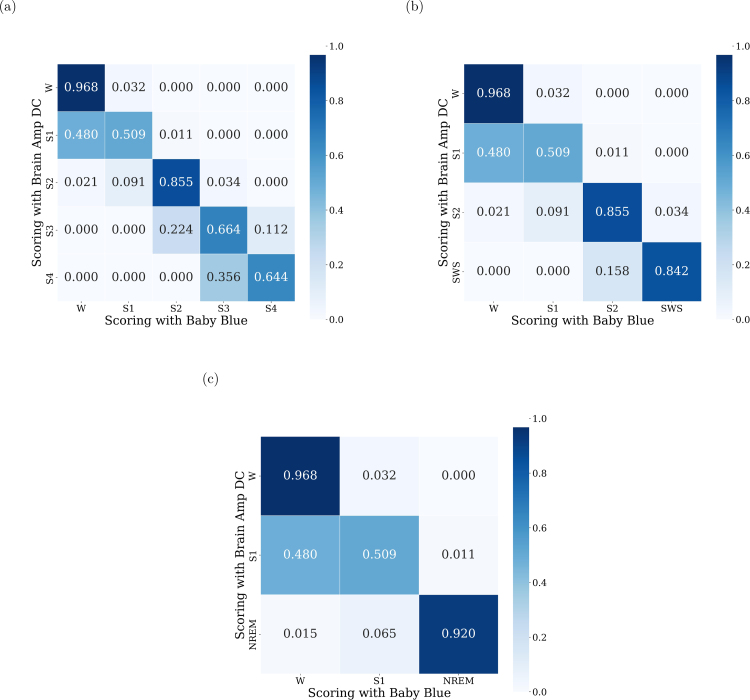
Normalized confusion matrix for the hypnograms between the signals obtained from both devices. The confusion matrix (a) displays predictions made by expert scorers, considering scoring based on BrainAmp DC records as true, while those classified with Baby Blue are taken as predictions. Stages are aligned with standard criteria such as wakefulness and stages 1–4. In matrix (b), stages 3 and 4 are considered as SWS, and in matrix (c), sleep stages 2, 3, and 4 are considered as NREM.

**Fig. 9:**
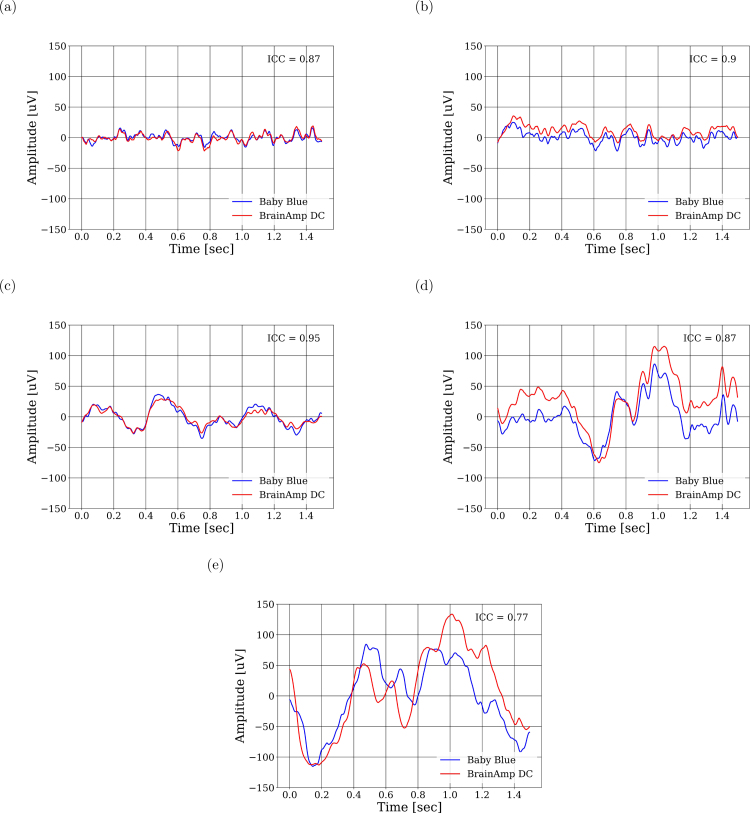
Comparison of signal fragments from all stages of sleep and intraclass correlations, (a) wakefulness, (b) S1, (c) S2, (d) S3 and (e) S4.

**Fig. 10:**
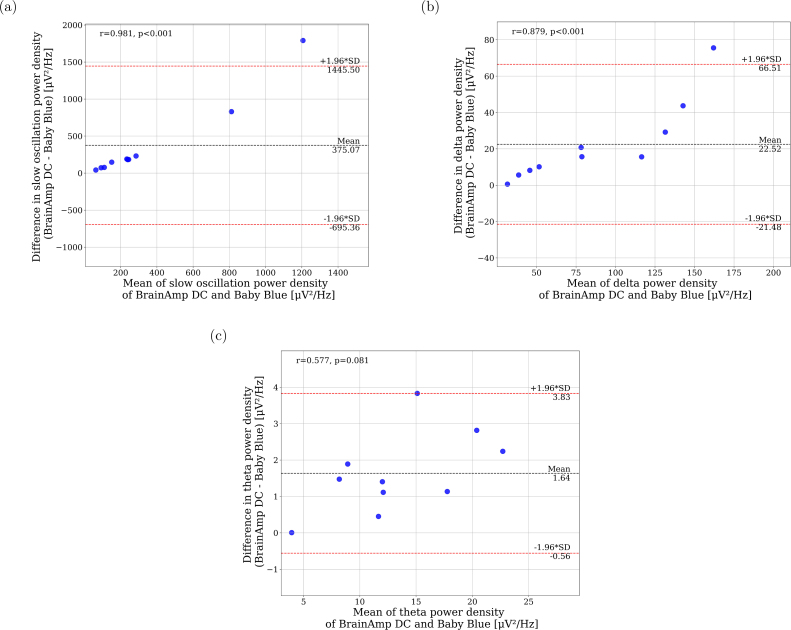
Bland–Altman plots of EEG power NREM sleep recorded at Cz. Mean EEG power from the BrainAmp DC and Baby Blue (y-axis) plotted against the difference in mean power (y-axis). (a) Slow Oscillation band, (b) Delta band, and (c) Theta band.

We conducted an analysis of the difference in mean power density using Bland-Altman plots. Our findings reveal that Baby Blue exhibits a negative bias when compared to BrainAmp DC for lower frequency bands (slow oscillation, delta, and theta bands) (Fig. 10). Given the high-pass filter of the AD8232 module, which has a cutoff frequency of 0.48 Hz, this result was anticipated. This characteristic implies a greater attenuation of low-frequency signals in comparison to the BrainAmp DC device.

We explored the effect of applying an additional second order band-pass filter of 0.48–0.53 Hz to the signal acquired with BrainAmp DC (the same values of the intrinsic filter of the Baby Blue’s AD8232 board), to confirm the impact of the intrinsic filter on the observed differences in low-frequency power density. After applying the filter, we found a decrement in the spectral power density in the signals acquire from BrainAmp DC, reducing the difference between devices (Supplementary Material, Figures S37 to S39). Thus, indicating that observed differences in low frequencies between devices are due to the presence of the intrinsic filter in Baby Blue.

**Fig. 11:**
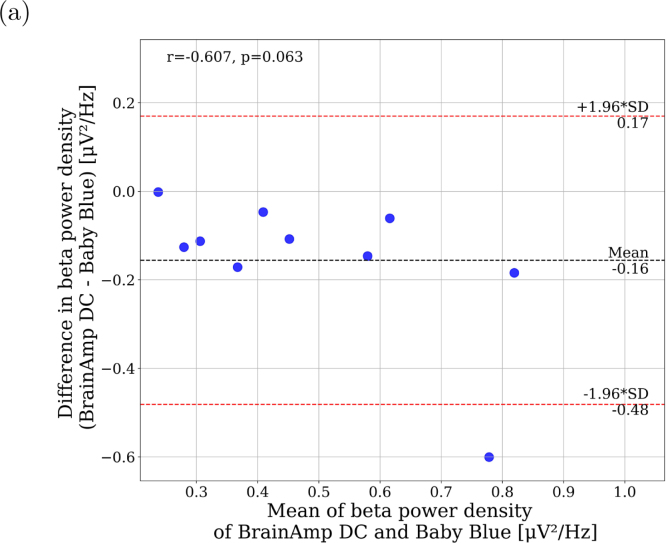
Bland–Altman plots of EEG power NREM sleep recorded at Cz. Mean EEG Beta power from the BrainAmp DC and Baby Blue (y-axis) plotted against the difference in mean power (y-axis).

Additionally, a discrepancy is observed in the beta frequency band for Baby Blue. The lack of sensors to measure EMG activity may lead to an inability to detect muscle movements that could generate artifacts in EEG signals, potentially increasing beta frequency power (Fig. 11). We attribute this variation to the artifact removal process applied to the BrainAmp DC signals, which could have reduced high-frequency bands in the EEG. We conducted additional analysis to investigate this hypothesis. Specifically, we repeated the power analysis without removing artifacts from the Brain-Amp DC recordings. Our observations indicate that the mean difference between devices in beta band power density is diminished (refer to Supplementary Material, Figure S36), implying that the inability to eliminate high-frequency activity resulting from artifacts (due to the absence of EMG signal) likely contributes to a discrepancy in power density within the beta band. In this comparison, Baby Blue exhibits higher beta band power density compared to BrainAmp DC.

When analyzing the Alpha, Slow, and Fast Spindles frequency bands, the mean difference approaches zero (see Fig. 12), indicating an agreement between the power measured by both devices. It is important to highlight that for all frequencies in each sleep stage, the majority of the mean difference between devices fall inside the confidence interval (see in Supplementary Material, Figures S1 to S35), indicating that the differences between both devices are consistent and do not show a significant bias in their variation.

Bland-Altman plots corresponding to this analysis can be found in the supplemental material ( https://doi.org/10.17605/OSF.IO/RJCHP).

**Fig. 12:**
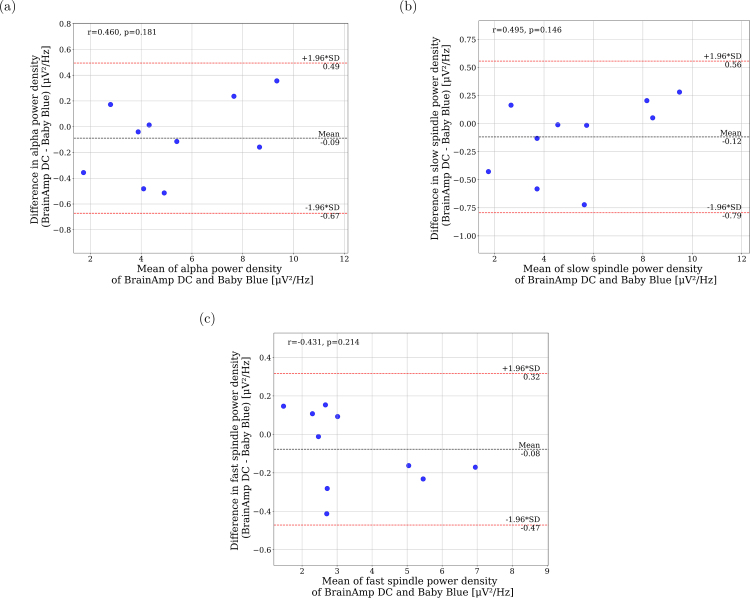
Bland–Altman plots of EEG power NREM sleep recorded at Cz. Mean EEG power from the BrainAmp DC and Baby Blue (y-axis) plotted against the difference in mean power (y-axis). (a) Alpha band, (b) Slow spindle band, and (c) Fast spindle band.

## 8 Conclusion

In this article, we demonstrate how to construct a low-cost, portable sleep monitoring device for acquiring EEG signals using only three electrodes. The decision to utilize just three electrodes simplifies the configuration process and significantly reduces costs. Nevertheless, our device offers scalability, allowing for the addition of more channels if required. In the present study, we confirm that the signal quality is comparable to that of other research-grade devices. Furthermore, the classification of NREM sleep stages was performed with high precision, demonstrating that our device enables accurate identification of total NREM sleep time and stages. Also, comparisons of power density between signals obtained from the BrainAmp DC and our device show coincidence. However, Baby Blue exhibits reduced power density in lower frequency bands, potentially resulting in inaccuracies when scoring sleep stages characterized by significant low-frequency oscillatory activity (e.g., S3 and S4). This also implies that the device is not interchangeable with other EEG devices within a study.

In conclusion, the low cost of device fabrication, straightforward configuration process, accurate sleep scoring in NREM sleep stages, and accordance between power density measurement, suggest that this device could be particularly useful for experiments requiring extensive data collection, and/or ecological studies conducted outside the laboratory, such as in subjects’ homes or at schools.

## Ethics statements

Participants signed a written informed consent approved by the Human Ethics Committee of the Universidad de Buenos Aires (Buenos Aires, Argentina) prior to participation in the study.

## CRediT authorship contribution statement

**Matías Rodolfo Pretel:** Writing – review & editing, Writing – original draft, Visualization, Validation, Methodology, Investigation, Formal analysis, Data curation, Conceptualization. **Vanessa Vidal:** Writing – review & editing, Validation, Software, Formal analysis. **Dante Kienigiel:** Writing – review & editing, Methodology. **Cecilia Forcato:** Writing – review & editing, Writing – original draft, Supervision, Resources, Project administration, Methodology, Funding acquisition, Conceptualization. **Rodrigo Ramele:** Writing – review & editing, Writing – original draft, Supervision, Resources, Methodology, Funding acquisition.

## Declaration of competing interest

The authors declare that they have no known competing financial interests or personal relationships that could have appeared to influence the work reported in this paper.

## Appendix A Supplementary data

MMC S1 The supplementary material includes Bland-Altman plots of EEG power across different frequency bands.
