# Supplementary material for: A low-cost and open-hardware portable 3-electrode sleep monitoring device
Source: HardwareX. 2024 Jul 6;19:e00553. doi: 10.1016/j.ohx.2024.e00553 (PMC11295469; doi:10.1016/j.ohx.2024.e00553)
Supplement: MMC S1 — The supplementary material includes Bland-Altman plots of EEG power across different frequency bands. [file mmc1.pdf]

A low-cost and open-hardware portable 3-electrode sleep monitoring device (supplementary material)

Matías Rodolfo Pretel[1]*, Vanessa Vidal[1,2], Dante Kienigiel[1], Cecilia Forcato[1†] & Rodrigo Ramele[3†]

[1]Laboratorio de Sueño y Memoria, Life Sciences Department, Instituto Tecnológico de Buenos Aires (ITBA), Buenos Aires, Argentina.
[2]Consejo Nacional de Investigaciones Científicas y Tecnológicas (CONICET), Buenos Aires, Argentina.
[3]Computer Engineering Department, Instituto Tecnológico de Buenos Aires (ITBA), Buenos Aires, Argentina.

[*]Corresponding author
E-mail address: mpretel@itba.edu.ar (M. Pretel)
[†] Contributed equally

1. Bland-Altman Plots

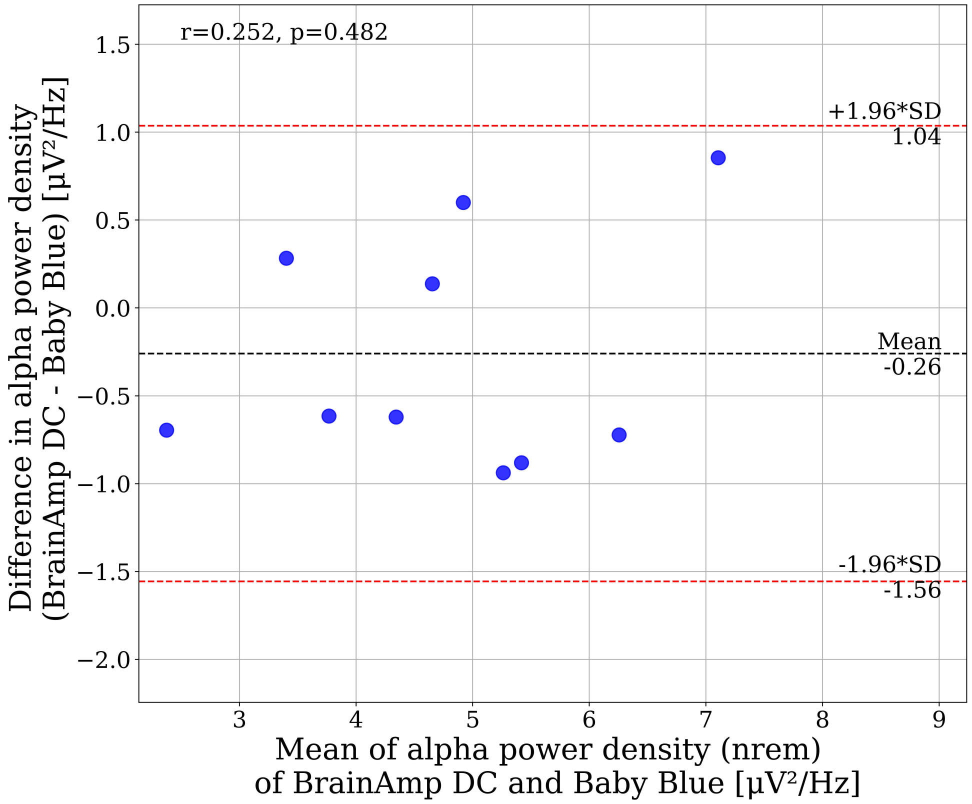

Figure 1: Bland–Altman plots of EEG power NREM sleep recorded at Cz. Mean EEG Alpha power from the BrainAmp DC and Baby Blue (y-axis) plotted against the difference in mean power (y-axis).

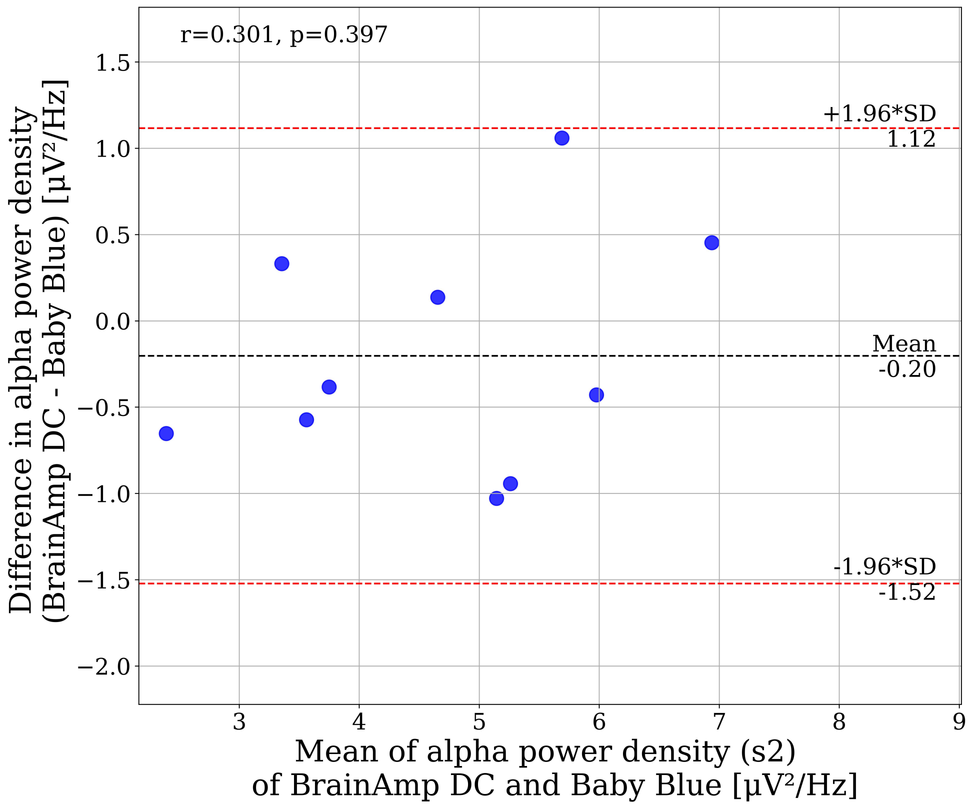

Figure 2: Bland–Altman plots of EEG power S2 sleep recorded at Cz. Mean EEG Alpha power from the BrainAmp DC and Baby Blue (y-axis) plotted against the difference in mean power (y-axis).

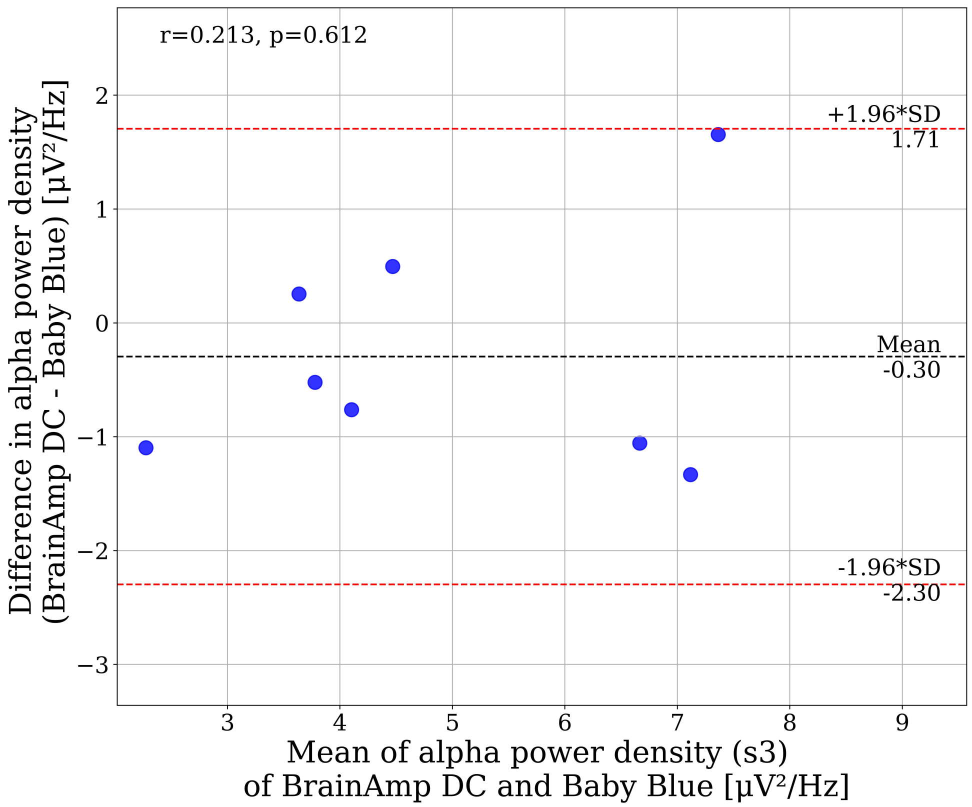

Figure 3: Bland–Altman plots of EEG power S3 sleep recorded at Cz. Mean EEG Alpha power from the BrainAmp DC and Baby Blue (y-axis) plotted against the difference in mean power (y-axis).

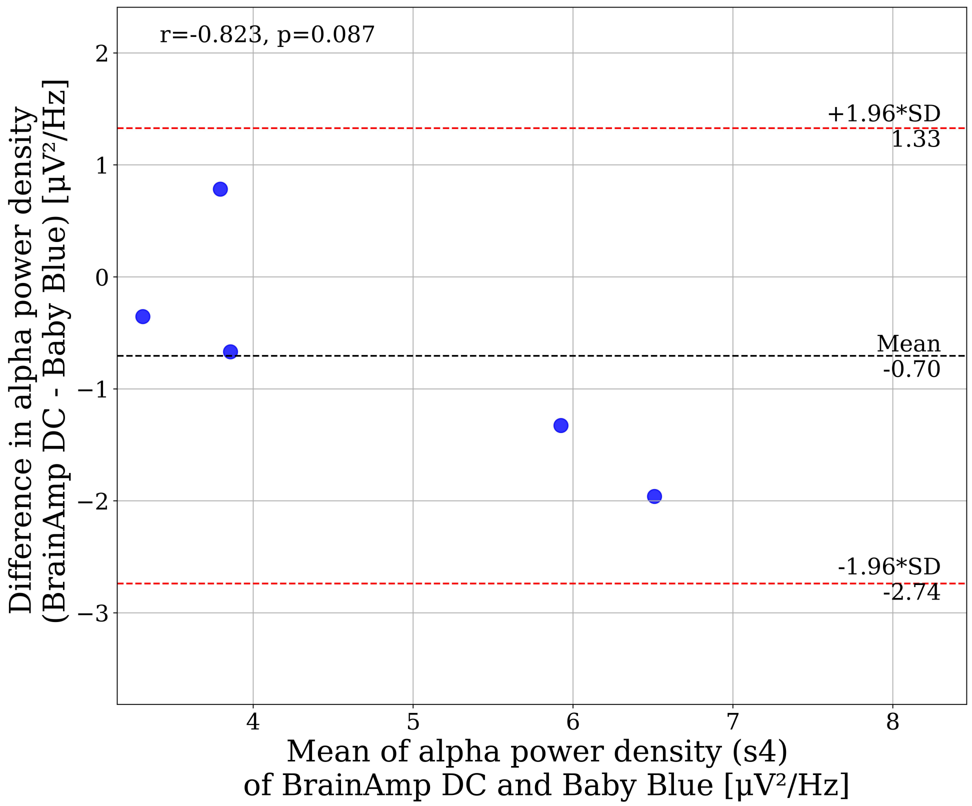

Figure 4: Bland–Altman plots of EEG power S4 sleep recorded at Cz. Mean EEG Alpha power from the BrainAmp DC and Baby Blue (y-axis) plotted against the difference in mean power (y-axis).

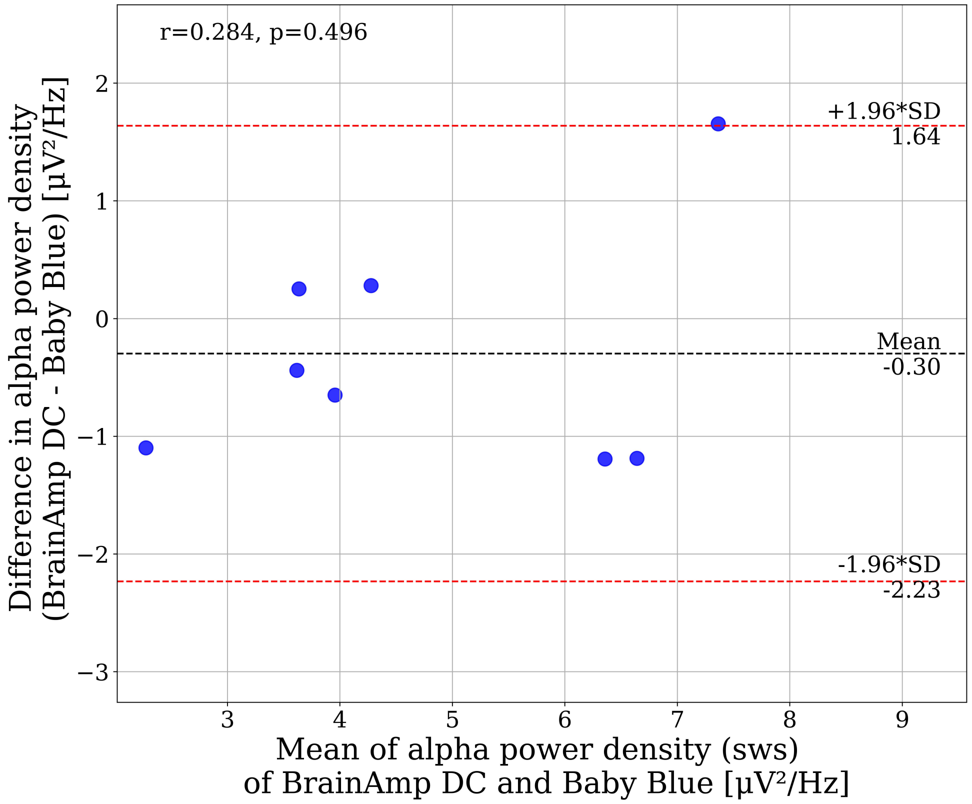

Figure 5: Bland–Altman plots of EEG power SWS recorded at Cz. Mean EEG Alpha power from the BrainAmp DC and Baby Blue (y-axis) plotted against the difference in mean power (y-axis).

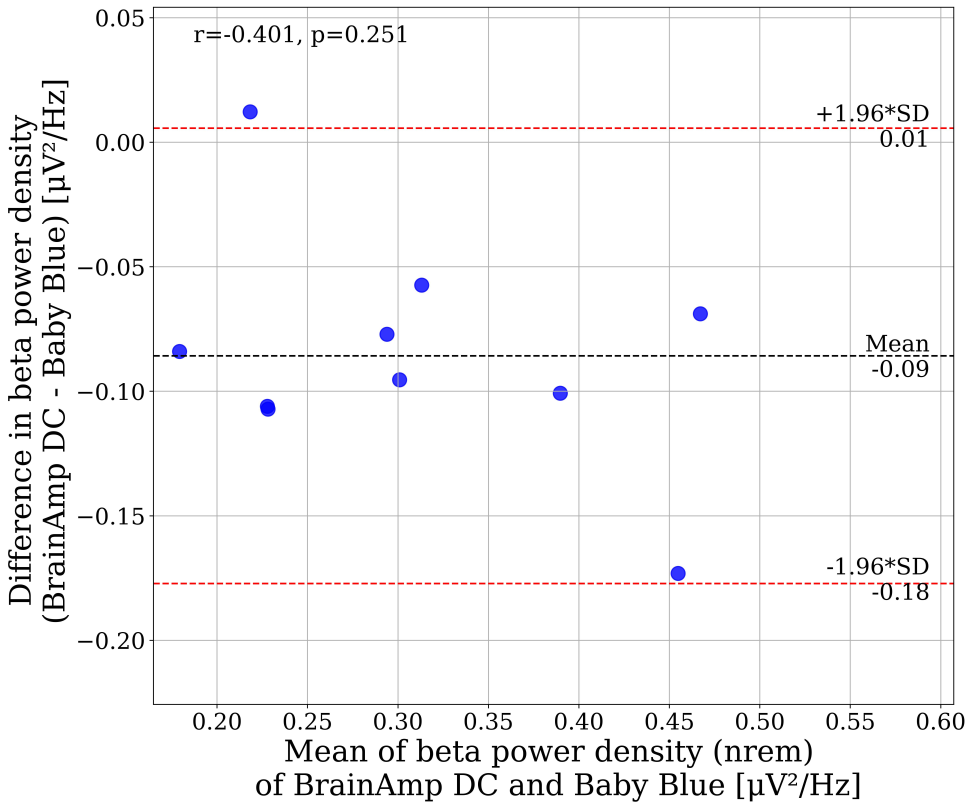

Figure 6: Bland–Altman plots of EEG power NREM sleep recorded at Cz. Mean EEG Beta power from the BrainAmp DC and Baby Blue (y-axis) plotted against the difference in mean power (y-axis).

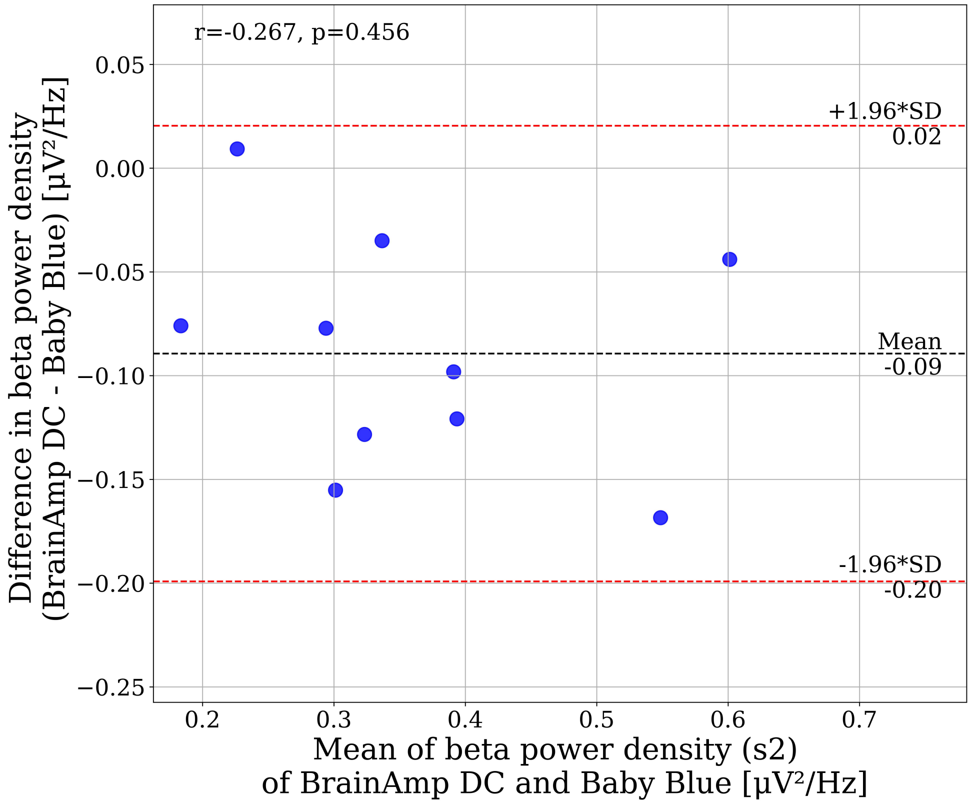

Figure 7: Bland–Altman plots of EEG power S2 sleep recorded at Cz. Mean EEG Beta power from the BrainAmp DC and Baby Blue (y-axis) plotted against the difference in mean power (y-axis).

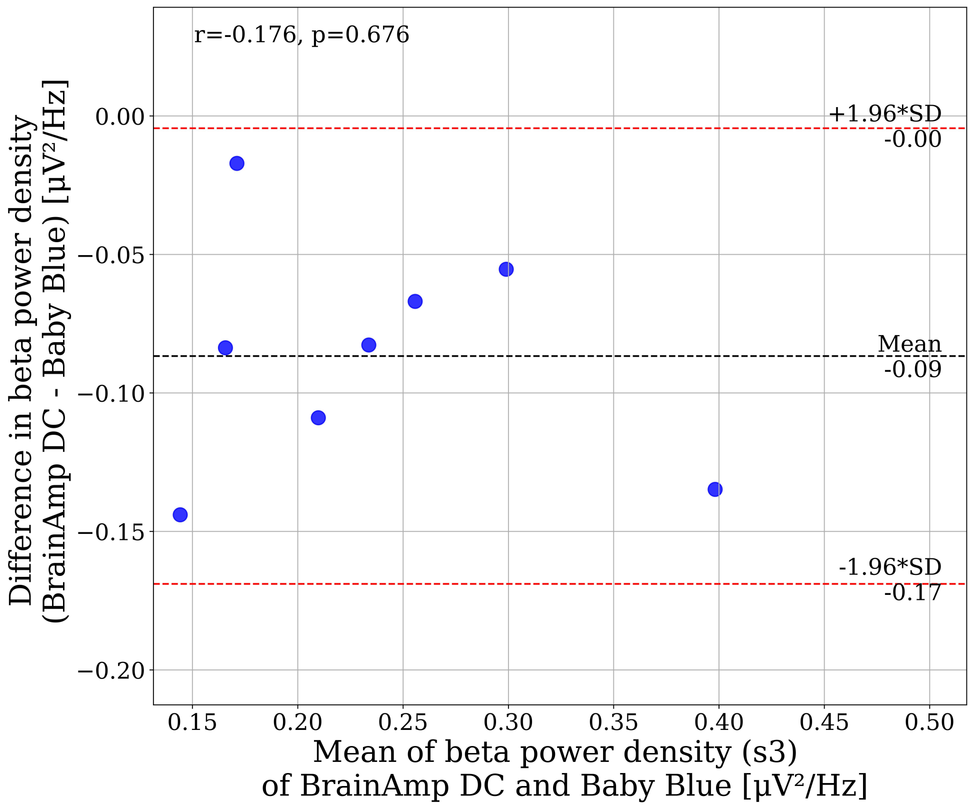

Figure 8: Bland–Altman plots of EEG power S3 sleep recorded at Cz. Mean EEG Beta power from the BrainAmp DC and Baby Blue (y-axis) plotted against the difference in mean power (y-axis).

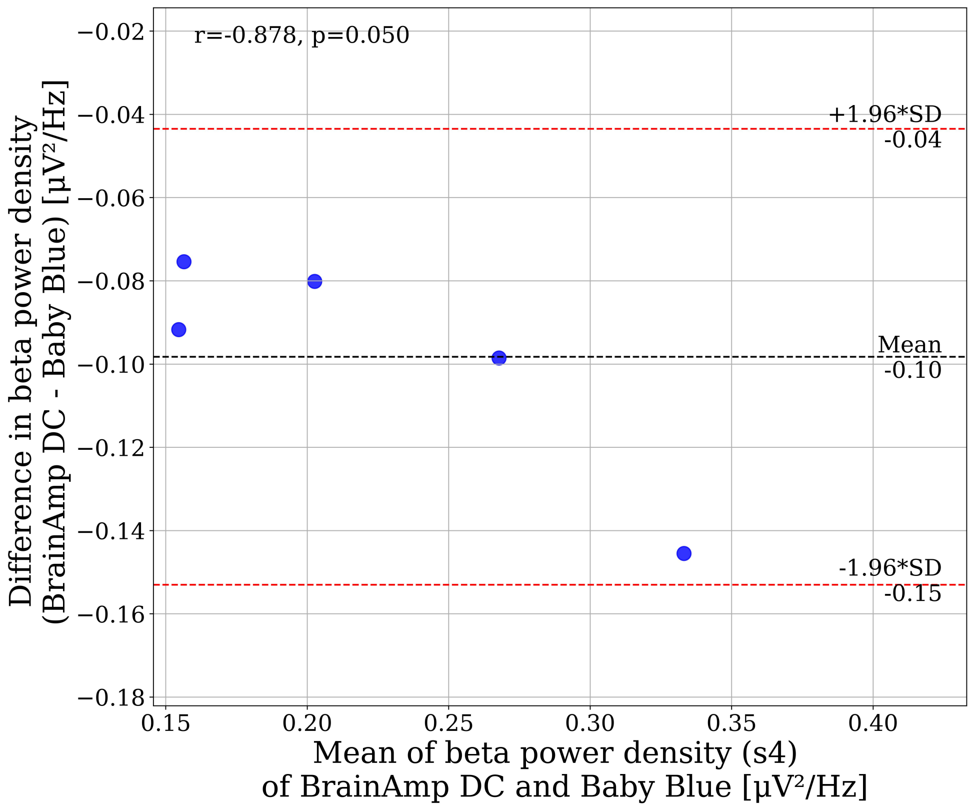

Figure 9: Bland–Altman plots of EEG power S4 sleep recorded at Cz. Mean EEG Beta power from the BrainAmp DC and Baby Blue (y-axis) plotted against the difference in mean power (y-axis).

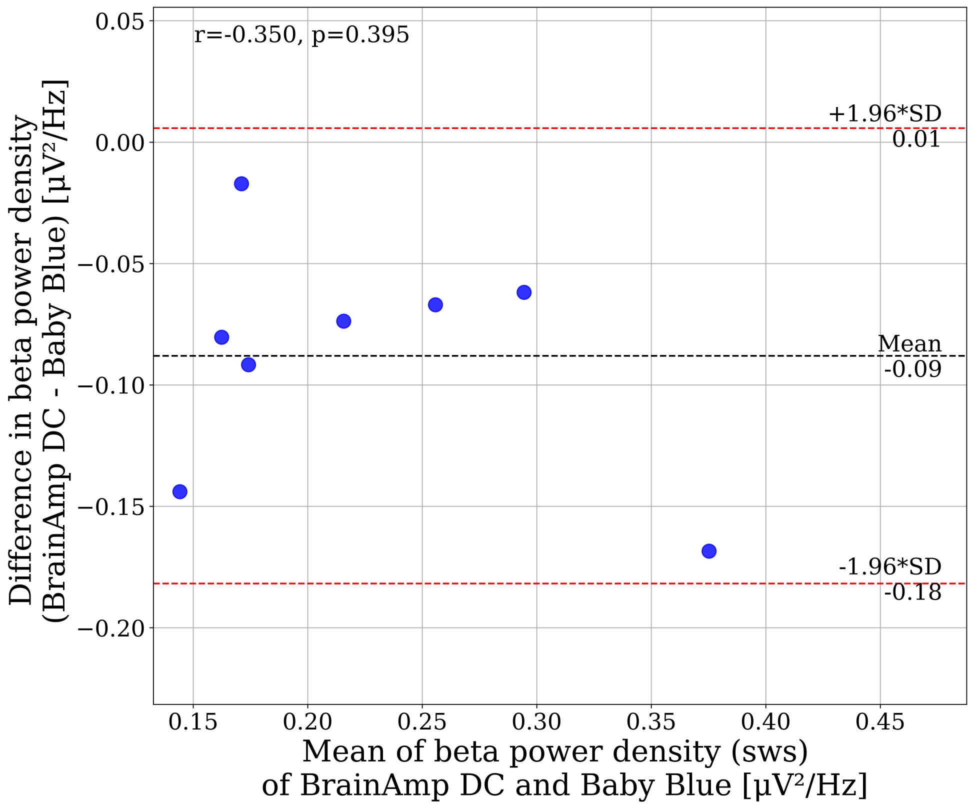

Figure 10: Bland–Altman plots of EEG power SWS recorded at Cz. Mean EEG Beta power from the BrainAmp DC and Baby Blue (y-axis) plotted against the difference in mean power (y-axis).

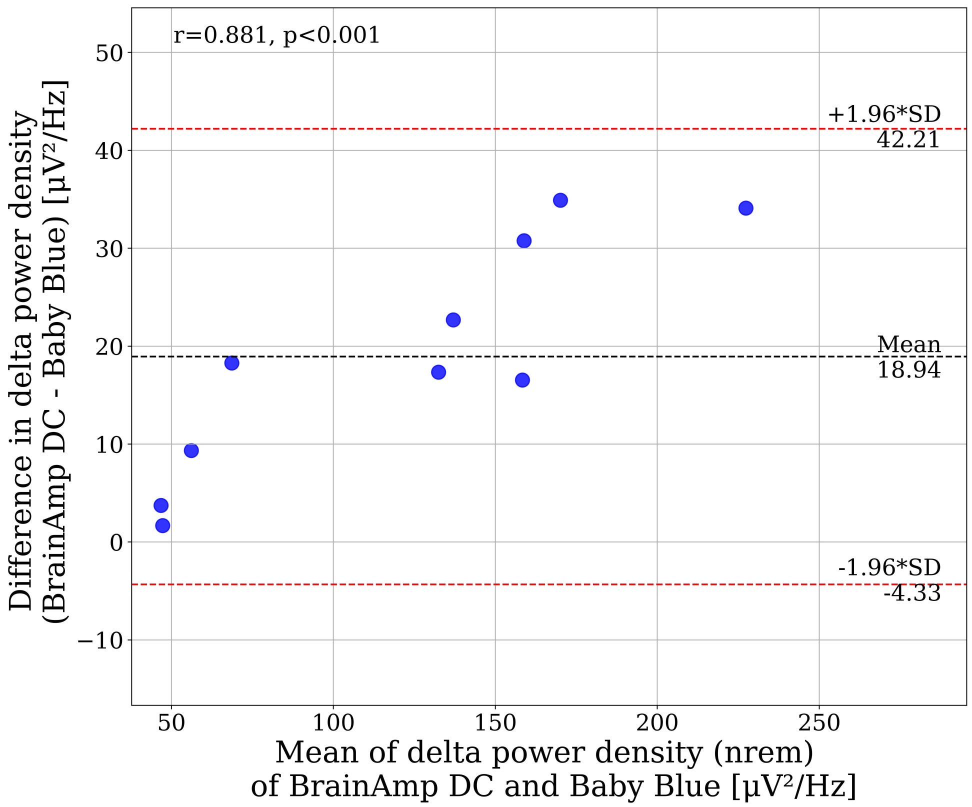

Figure 11: Bland–Altman plots of EEG power NREM sleep recorded at Cz. Mean EEG Delta power from the BrainAmp DC and Baby Blue (y-axis) plotted against the difference in mean power (y-axis).

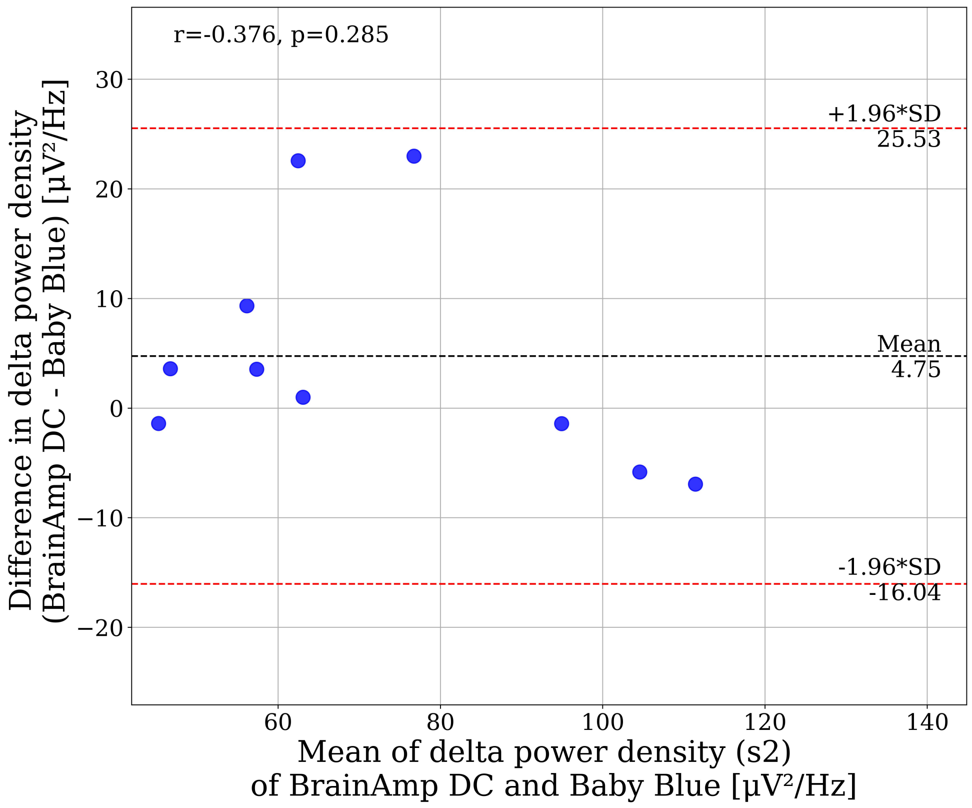

Figure 12: Bland–Altman plots of EEG power S2 sleep recorded at Cz. Mean EEG Delta power from the BrainAmp DC and Baby Blue (y-axis) plotted against the difference in mean power (y-axis).

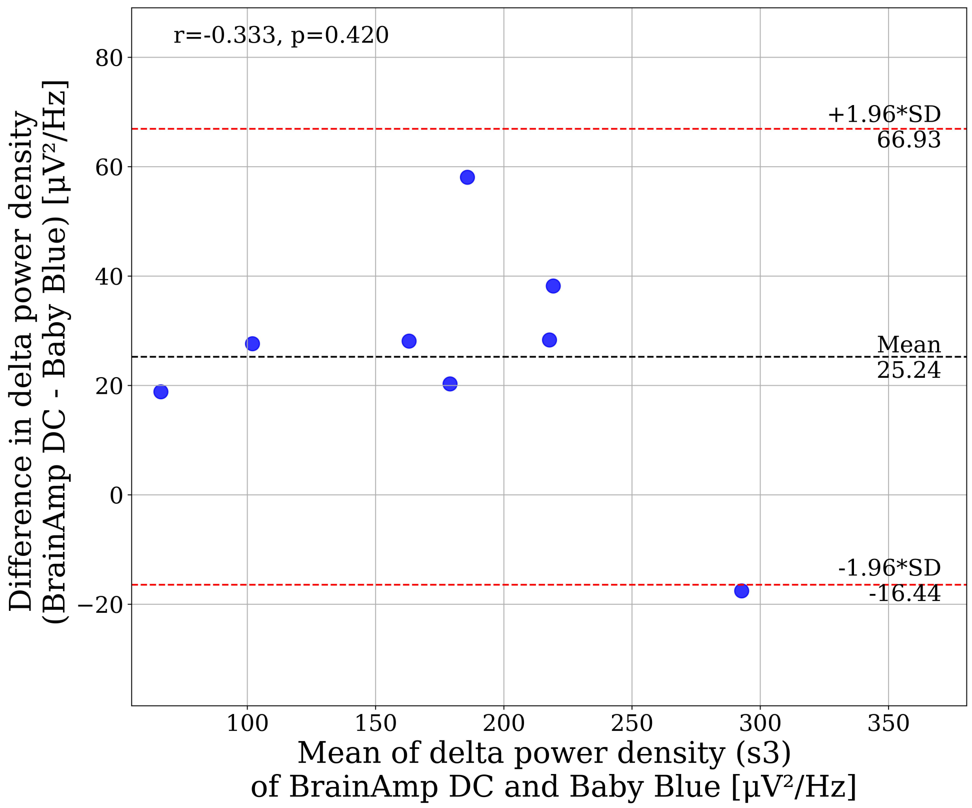

Figure 13: Bland–Altman plots of EEG power S3 sleep recorded at Cz. Mean EEG Delta power from the BrainAmp DC and Baby Blue (y-axis) plotted against the difference in mean power (y-axis).

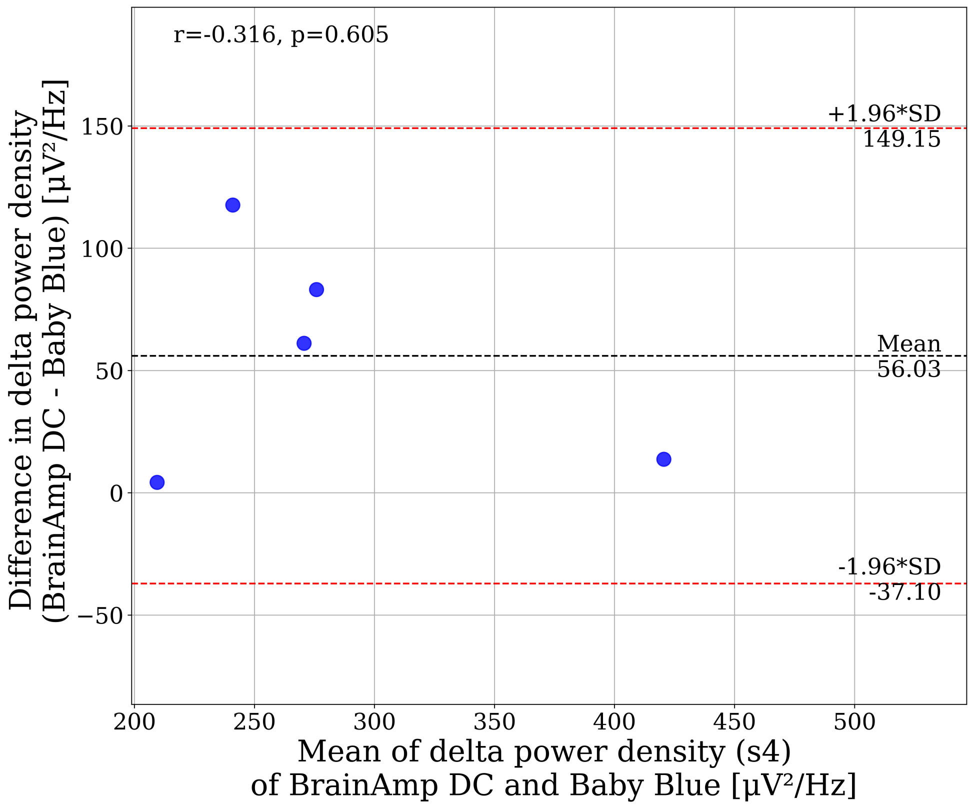

Figure 14: Bland–Altman plots of EEG power S4 sleep recorded at Cz. Mean EEG Delta power from the BrainAmp DC and Baby Blue (y-axis) plotted against the difference in mean power (y-axis).

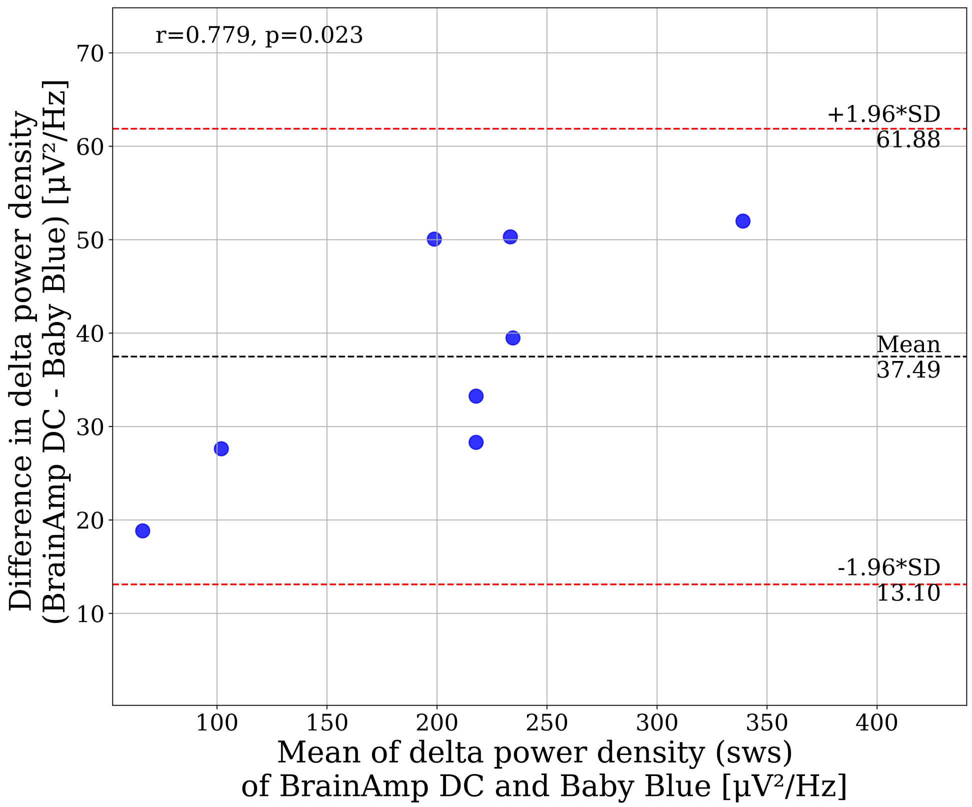

Figure 15: Bland–Altman plots of EEG power SWS recorded at Cz. Mean EEG Delta power from the BrainAmp DC and Baby Blue (y-axis) plotted against the difference in mean power (y-axis).

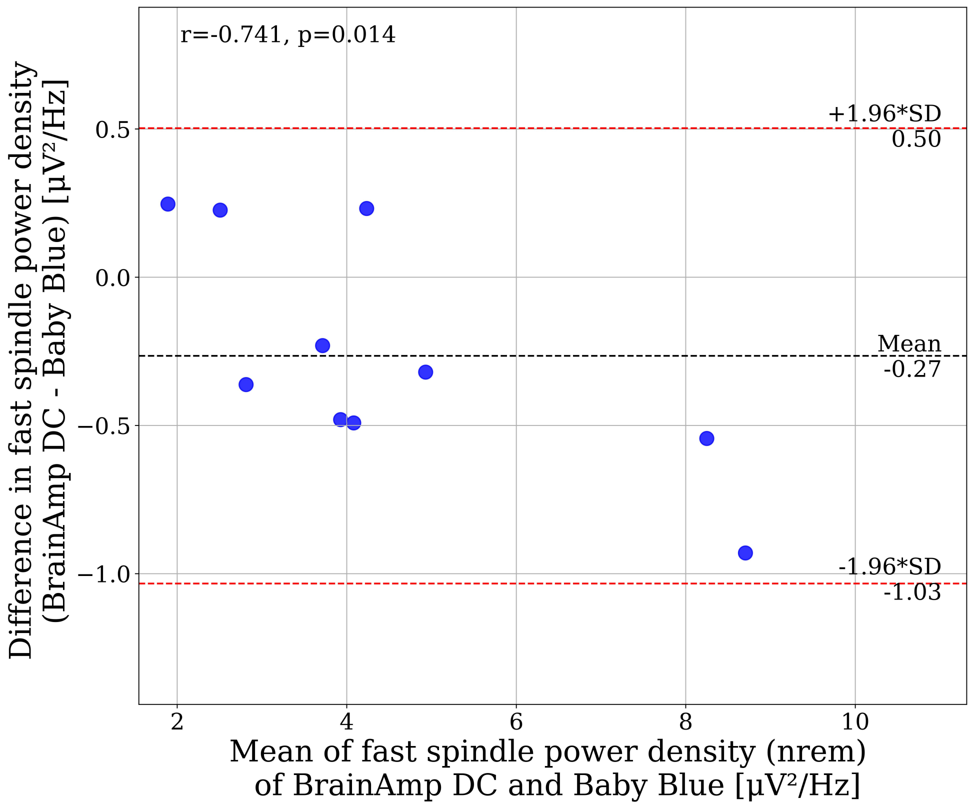

Figure 16: Bland–Altman plots of EEG power NREM sleep recorded at Cz. Mean EEG Fast Spindle power from the BrainAmp DC and Baby Blue (y-axis) plotted against the difference in mean power (y-axis).

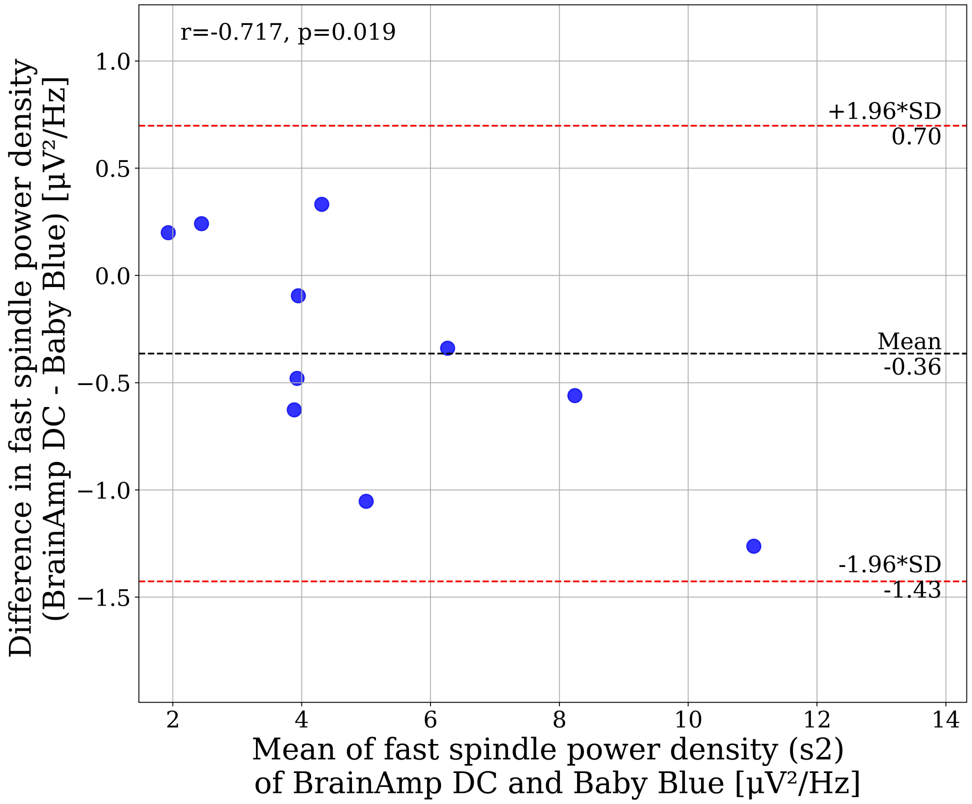

Figure 17: Bland–Altman plots of EEG power S2 sleep recorded at Cz. Mean EEG Fast Spindle power from the BrainAmp DC and Baby Blue (y-axis) plotted against the difference in mean power (y-axis).

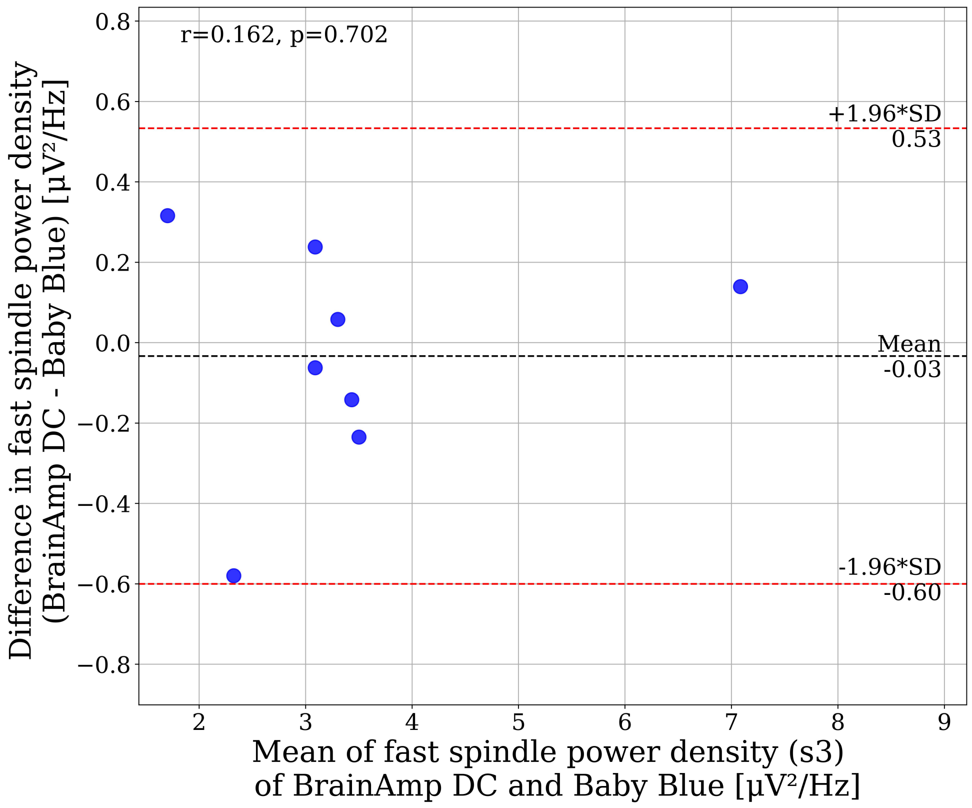

Figure 18: Bland–Altman plots of EEG power S3 sleep recorded at Cz. Mean EEG Fast Spindle power from the BrainAmp DC and Baby Blue (y-axis) plotted against the difference in mean power (y-axis).

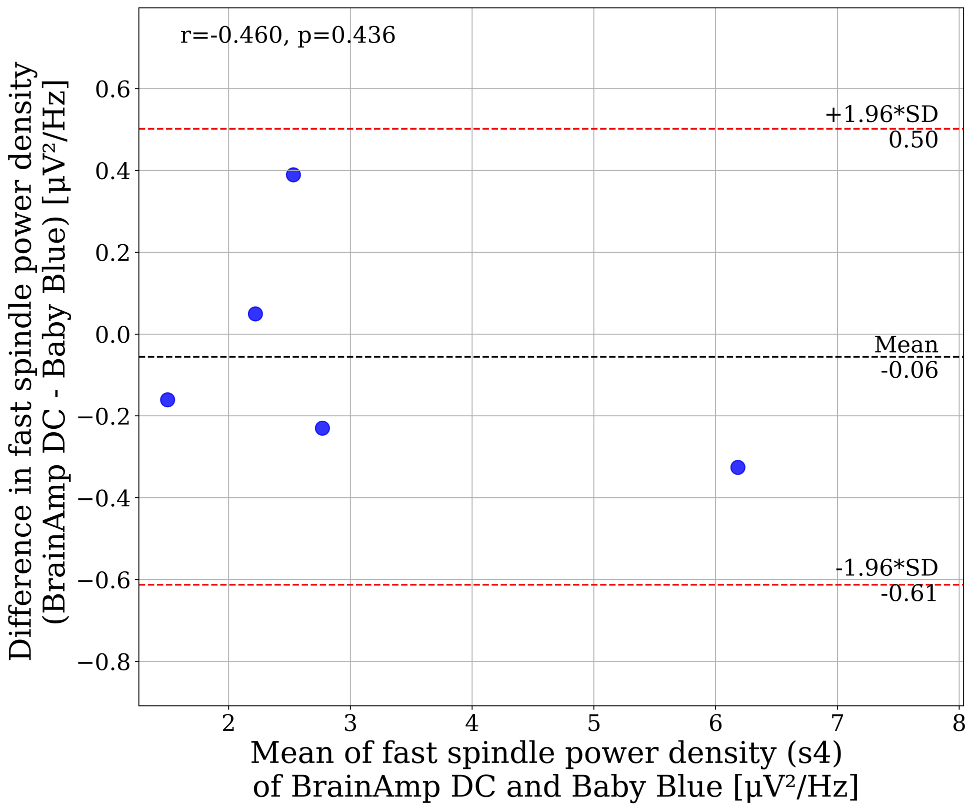

Figure 19: Bland–Altman plots of EEG power S4 sleep recorded at Cz. Mean EEG Fast Spindle power from the BrainAmp DC and Baby Blue (y-axis) plotted against the difference in mean power (y-axis).

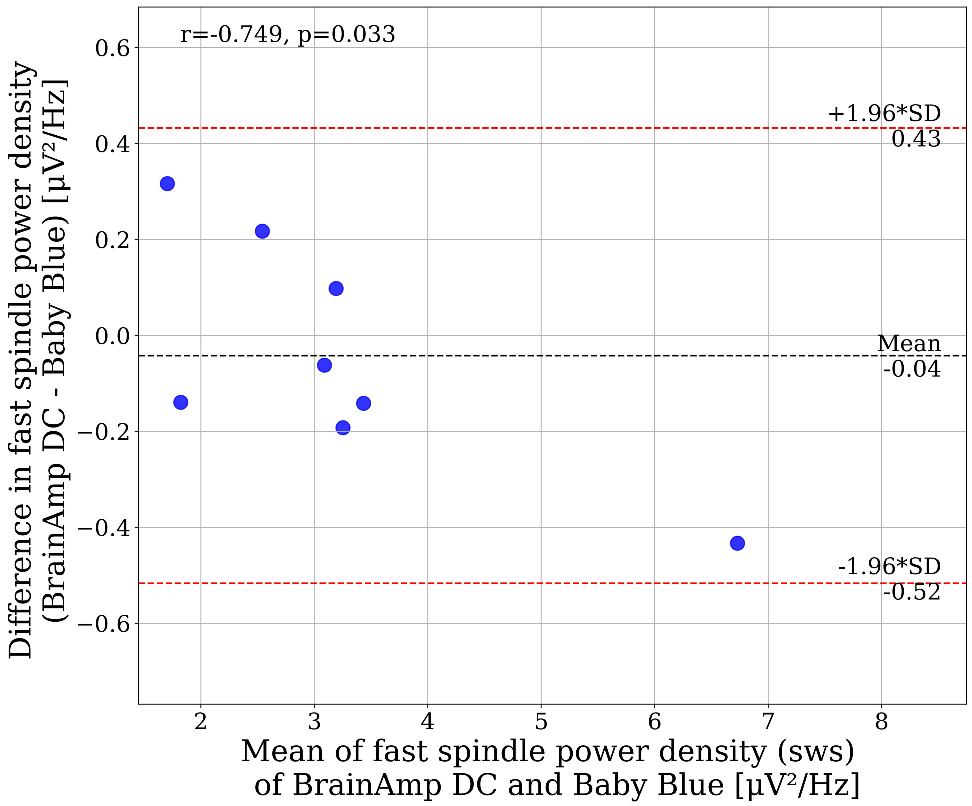

Figure 20: Bland–Altman plots of EEG power SWS recorded at Cz. Mean EEG Fast Spindle power from the BrainAmp DC and Baby Blue (y-axis) plotted against the difference in mean power (y-axis).

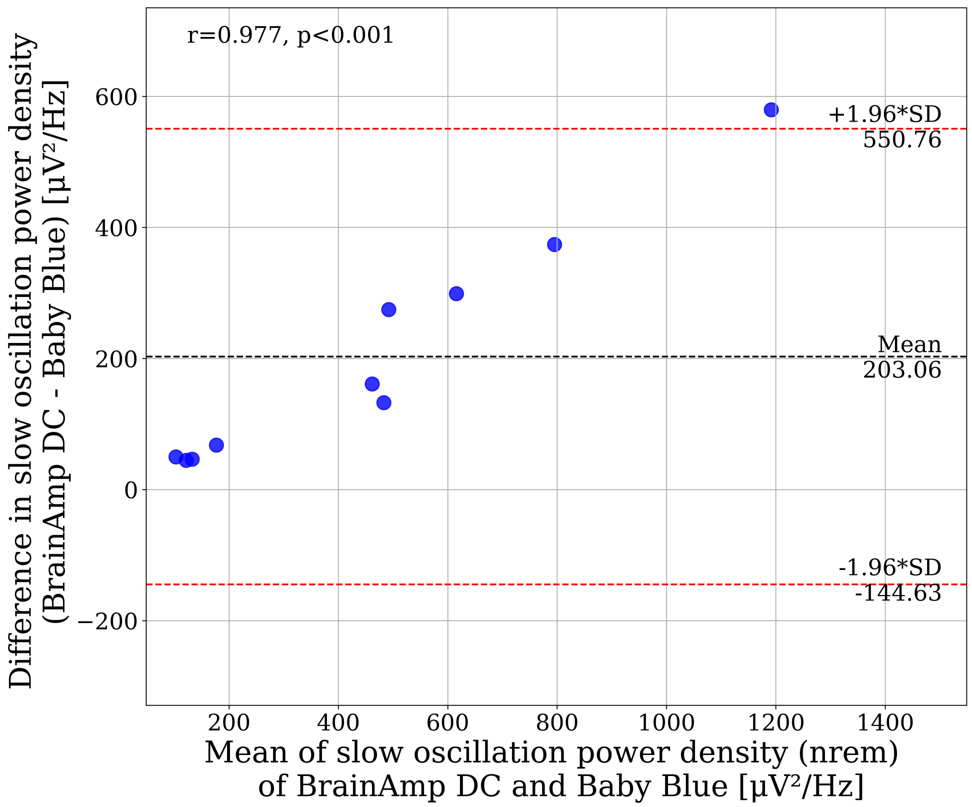

Figure 21: Bland–Altman plots of EEG power NREM sleep recorded at Cz. Mean EEG Slow Oscillation power from the BrainAmp DC and Baby Blue (y-axis) plotted against the difference in mean power (y-axis).

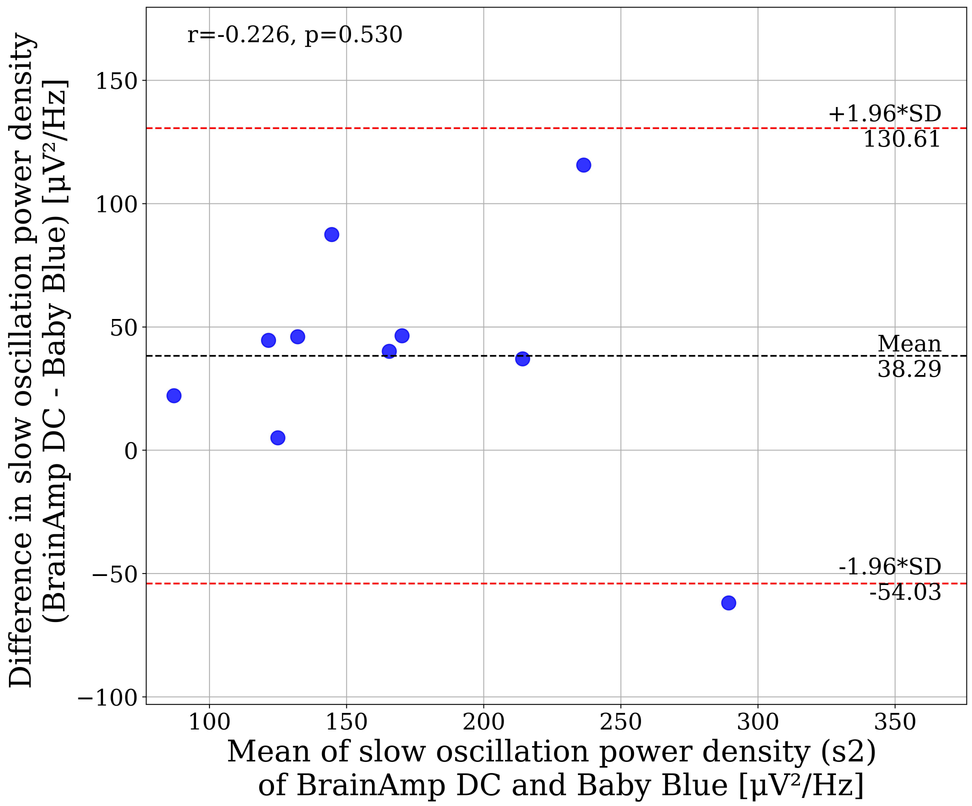

Figure 22: Bland–Altman plots of EEG power S2 sleep recorded at Cz. Mean EEG Slow Oscillation power from the BrainAmp DC and Baby Blue (y-axis) plotted against the difference in mean power (y-axis).

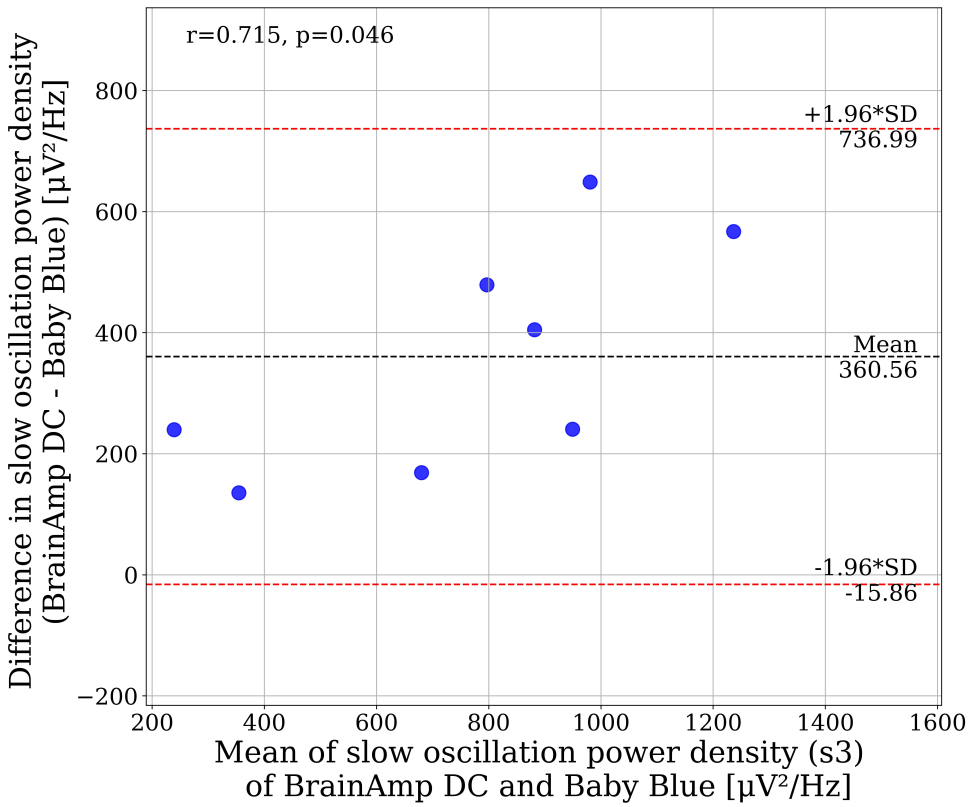

Figure 23: Bland–Altman plots of EEG power S3 sleep recorded at Cz. Mean EEG Slow Oscillation power from the BrainAmp DC and Baby Blue (y-axis) plotted against the difference in mean power (y-axis).

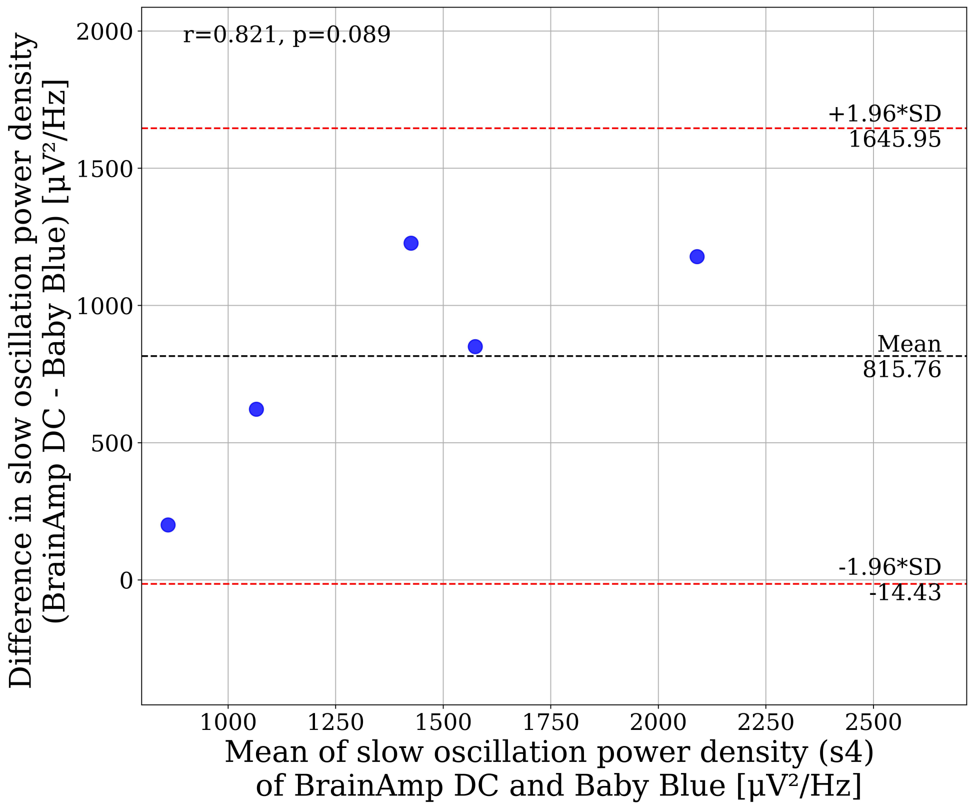

Figure 24: Bland–Altman plots of EEG power S4 sleep recorded at Cz. Mean EEG Slow Oscillation power from the BrainAmp DC and Baby Blue (y-axis) plotted against the difference in mean power (y-axis).

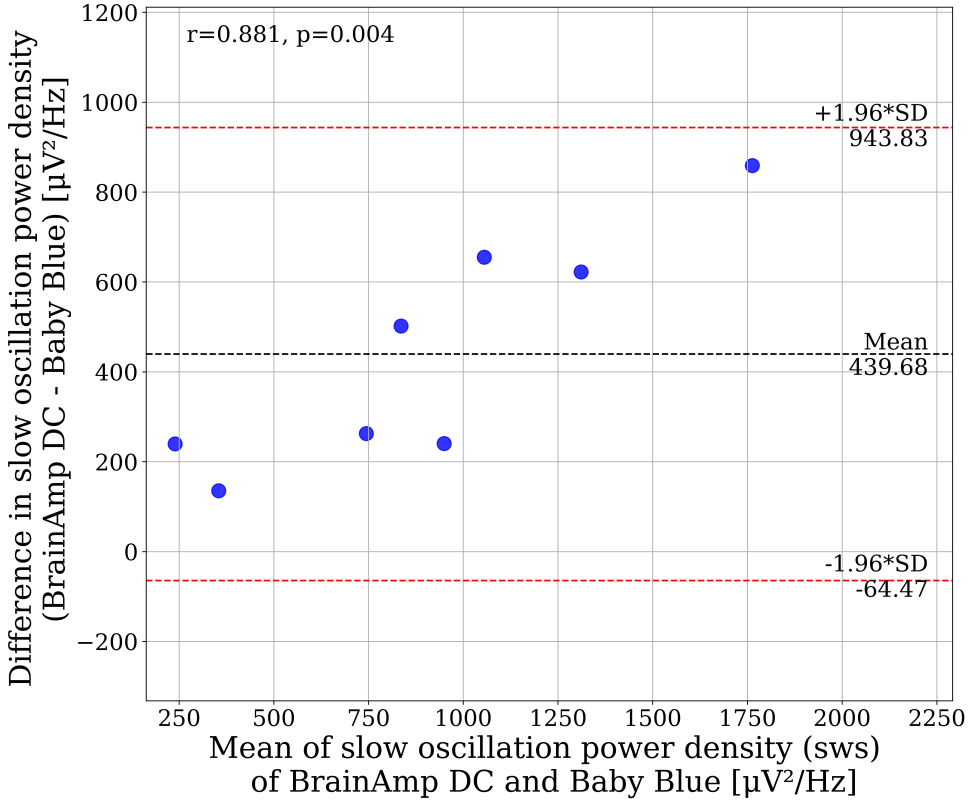

Figure 25: Bland–Altman plots of EEG power SWS recorded at Cz. Mean EEG Slow Oscillation power from the BrainAmp DC and Baby Blue (y-axis) plotted against the difference in mean power (y-axis).

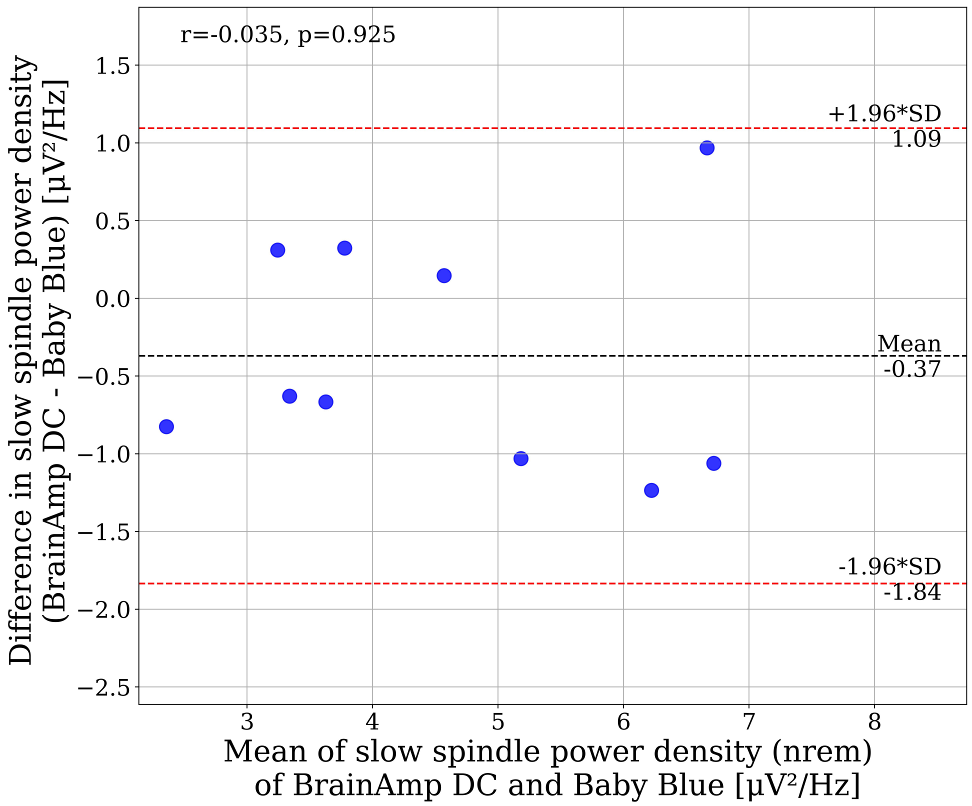

Figure 26: Bland–Altman plots of EEG power NREM sleep recorded at Cz. Mean EEG Slow Spindle power from the BrainAmp DC and Baby Blue (y-axis) plotted against the difference in mean power (y-axis).

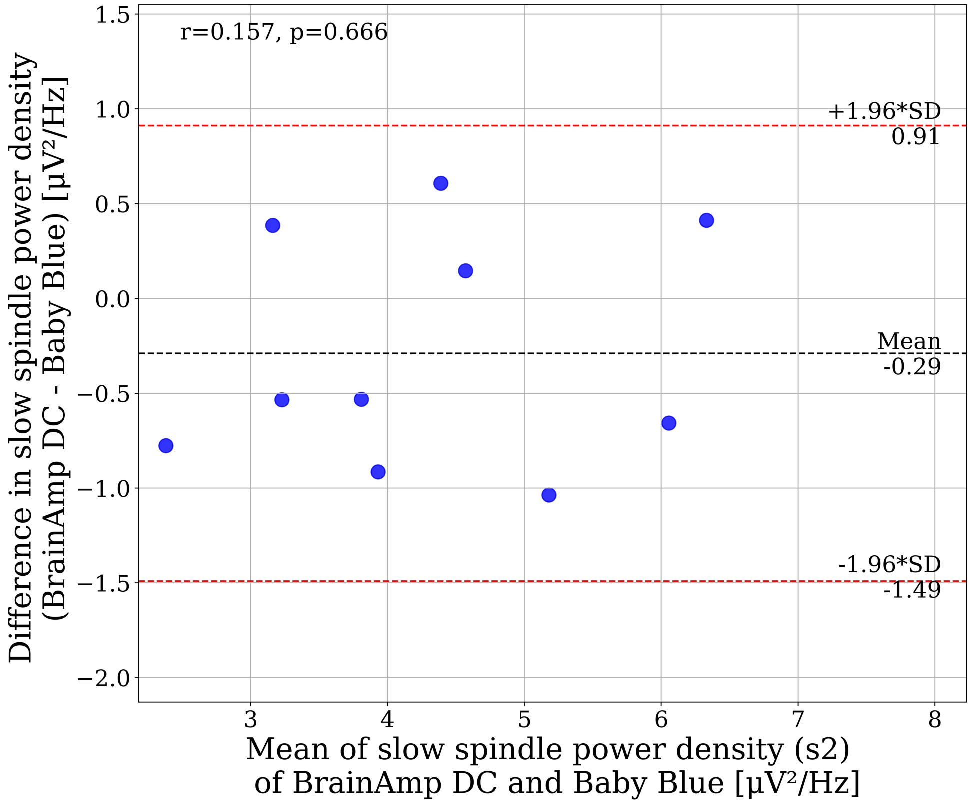

Figure 27: Bland–Altman plots of EEG power S2 sleep recorded at Cz. Mean EEG Slow Spindle power from the BrainAmp DC and Baby Blue (y-axis) plotted against the difference in mean power (y-axis).

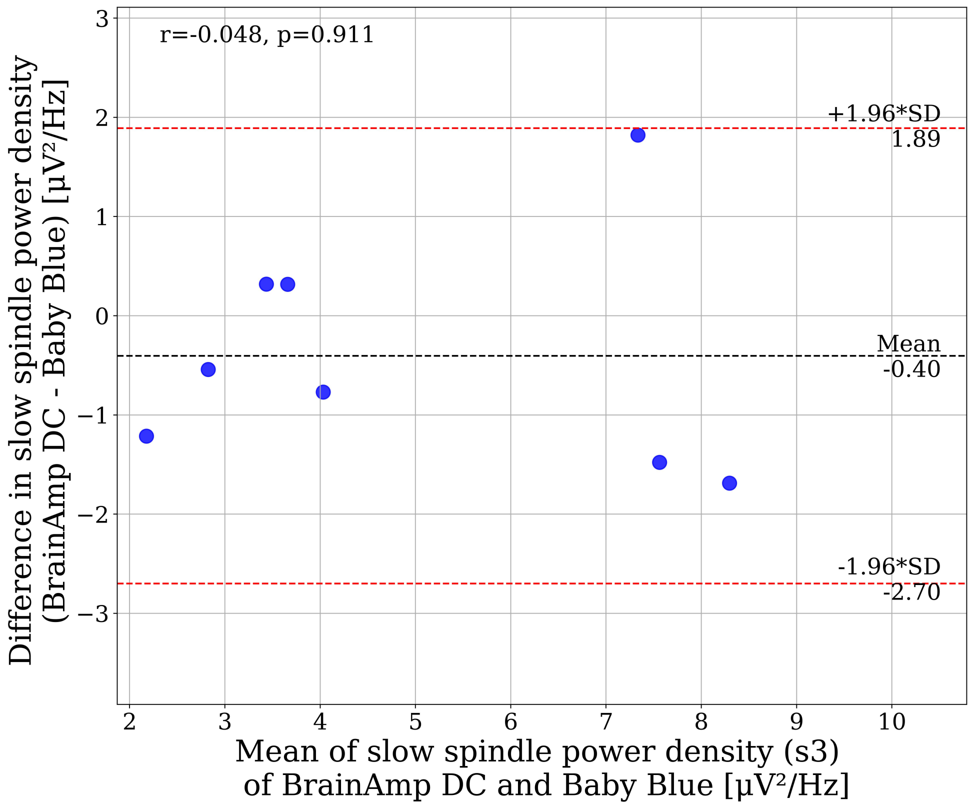

Figure 28: Bland–Altman plots of EEG power S2 sleep recorded at Cz. Mean EEG Slow Spindle power from the BrainAmp DC and Baby Blue (y-axis) plotted against the difference in mean power (y-axis).

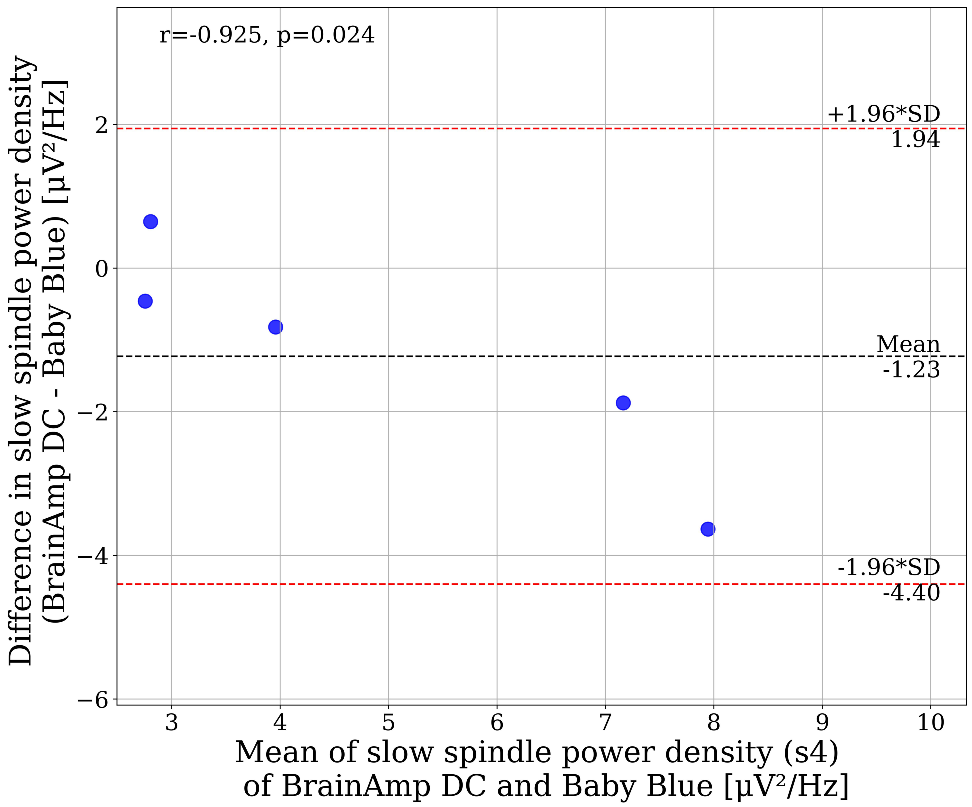

Figure 29: Bland–Altman plots of EEG power S4 sleep recorded at Cz. Mean EEG Slow Spindle power from the BrainAmp DC and Baby Blue (y-axis) plotted against the difference in mean power (y-axis).

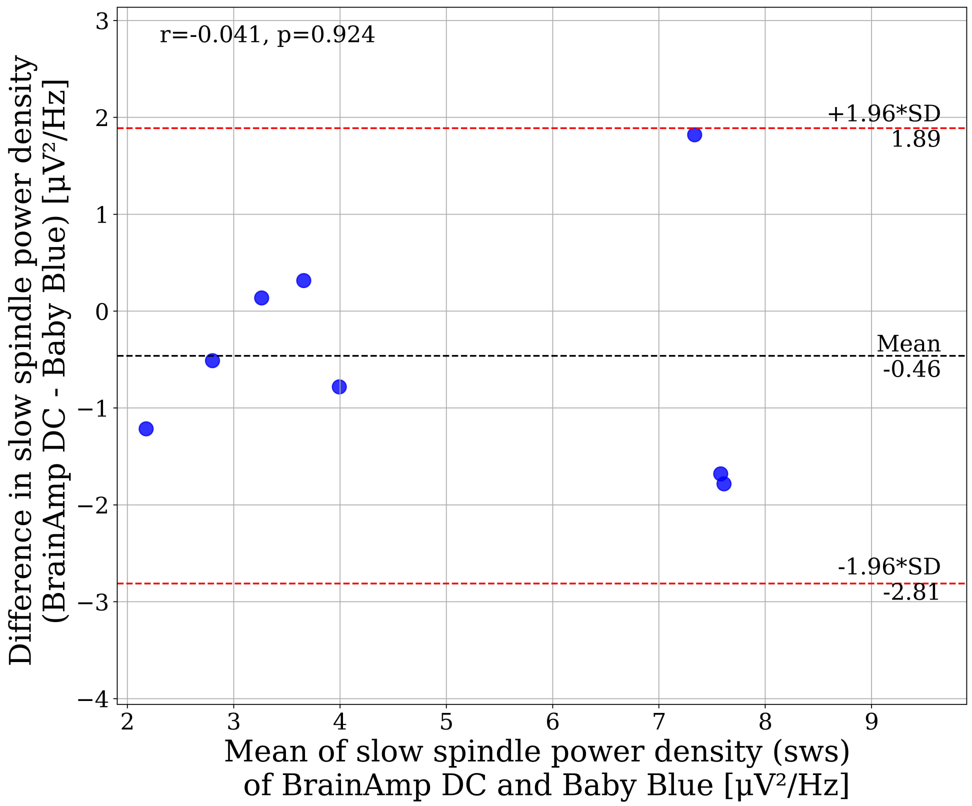

Figure 30: Bland–Altman plots of EEG power SWS recorded at Cz. Mean EEG Slow Spindle power from the BrainAmp DC and Baby Blue (y-axis) plotted against the difference in mean power (y-axis).

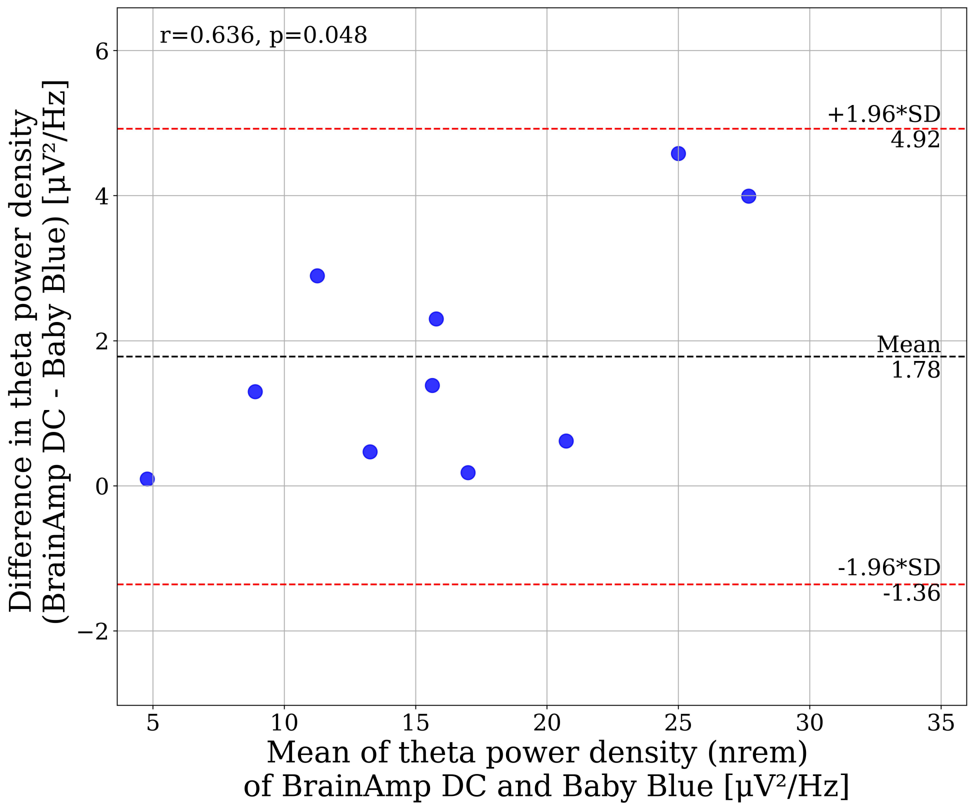

Figure 31: Bland–Altman plots of EEG power NREM sleep recorded at Cz. Mean EEG Theta power from the BrainAmp DC and Baby Blue (y-axis) plotted against the difference in mean power (y-axis).

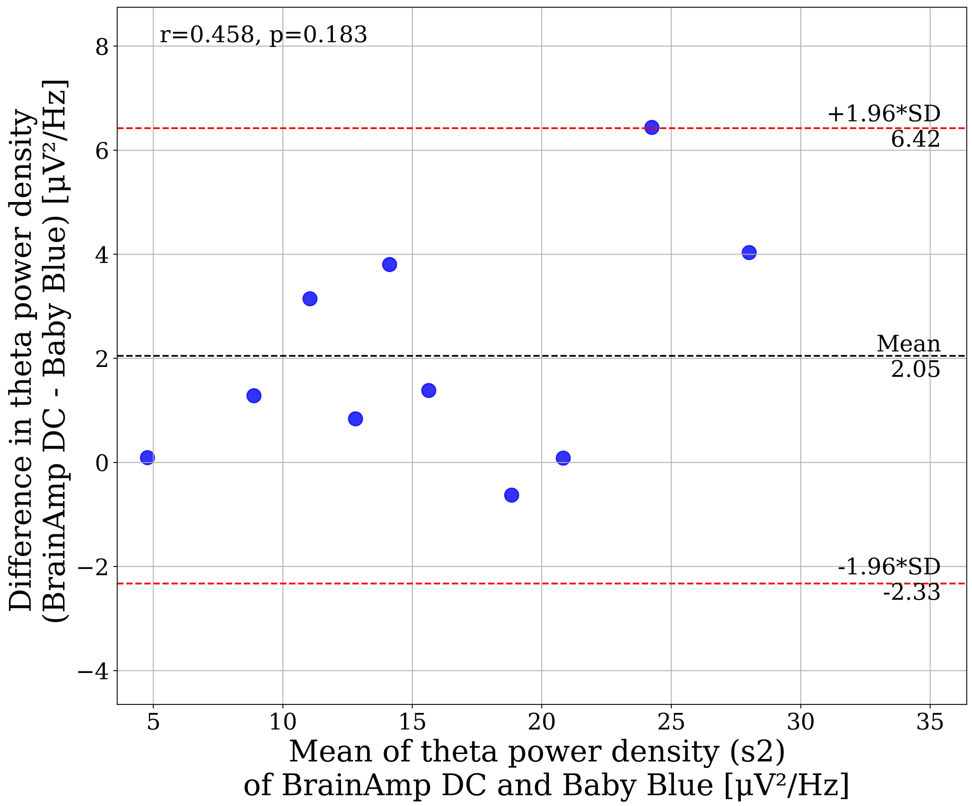

Figure 32: Bland–Altman plots of EEG power S2 sleep recorded at Cz. Mean EEG Theta power from the BrainAmp DC and Baby Blue (y-axis) plotted against the difference in mean power (y-axis).

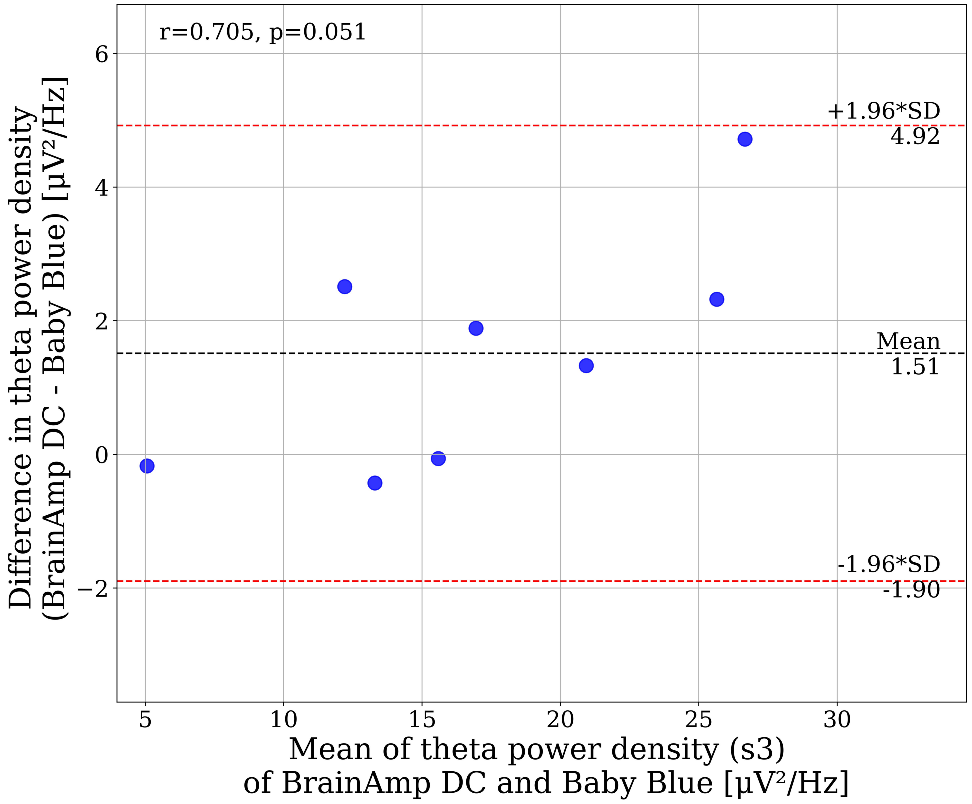

Figure 33: Bland–Altman plots of EEG power S3 sleep recorded at Cz. Mean EEG Theta power from the BrainAmp DC and Baby Blue (y-axis) plotted against the difference in mean power (y-axis).

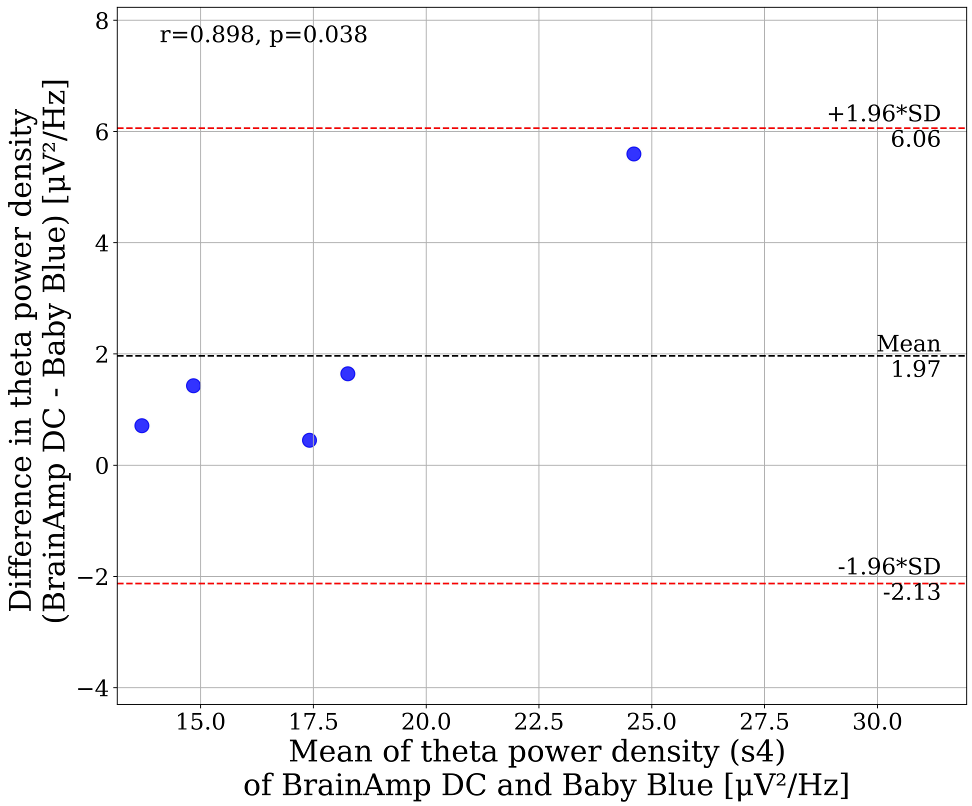

Figure 34: Bland–Altman plots of EEG power s4 sleep recorded at Cz. Mean EEG Theta power from the BrainAmp DC and Baby Blue (y-axis) plotted against the difference in mean power (y-axis).

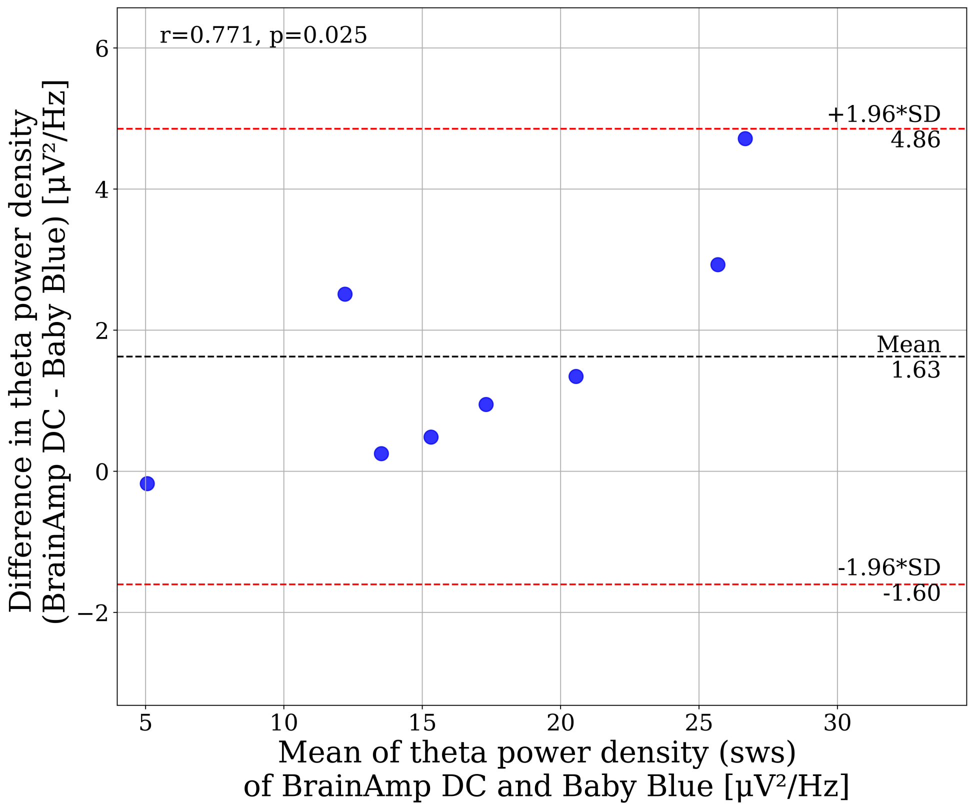

Figure 35: Bland–Altman plots of EEG power SWS recorded at Cz. Mean EEG Theta power from the BrainAmp DC and Baby Blue (y-axis) plotted against the difference in mean power (y-axis).

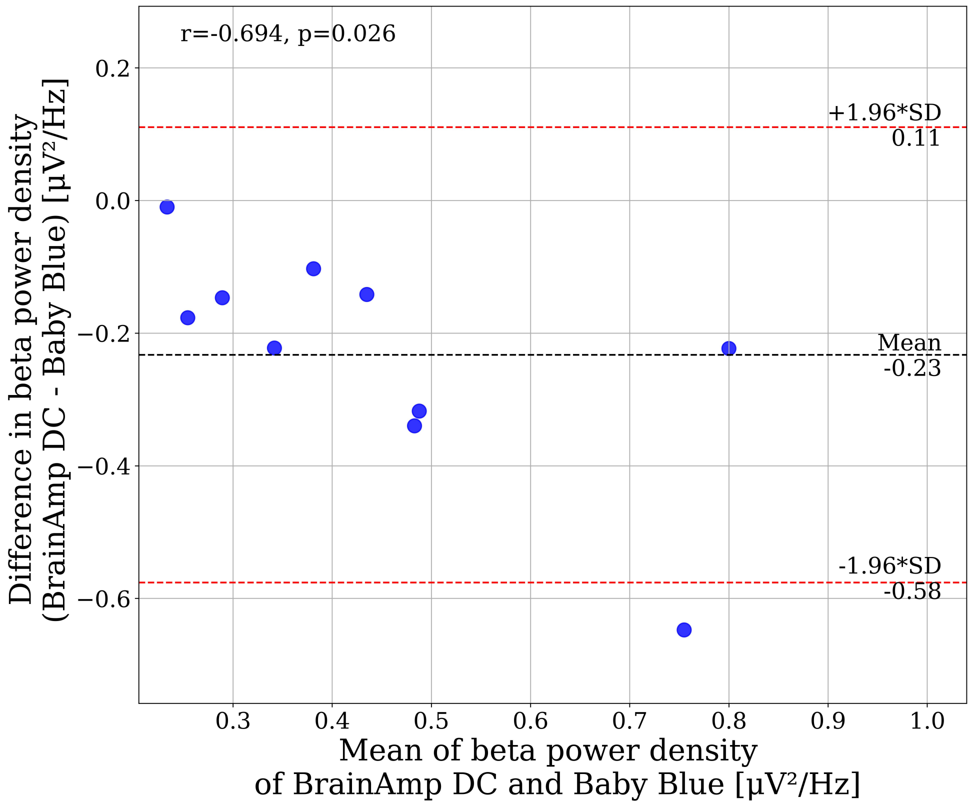

Figure 36: Bland–Altman plots of EEG power NREM sleep recorded at Cz. Mean EEG Beta power from the BrainAmp DC and Baby Blue (y-axis) plotted against the difference in mean power (y-axis). Artifacts from BrainAmp DC signals were removed.

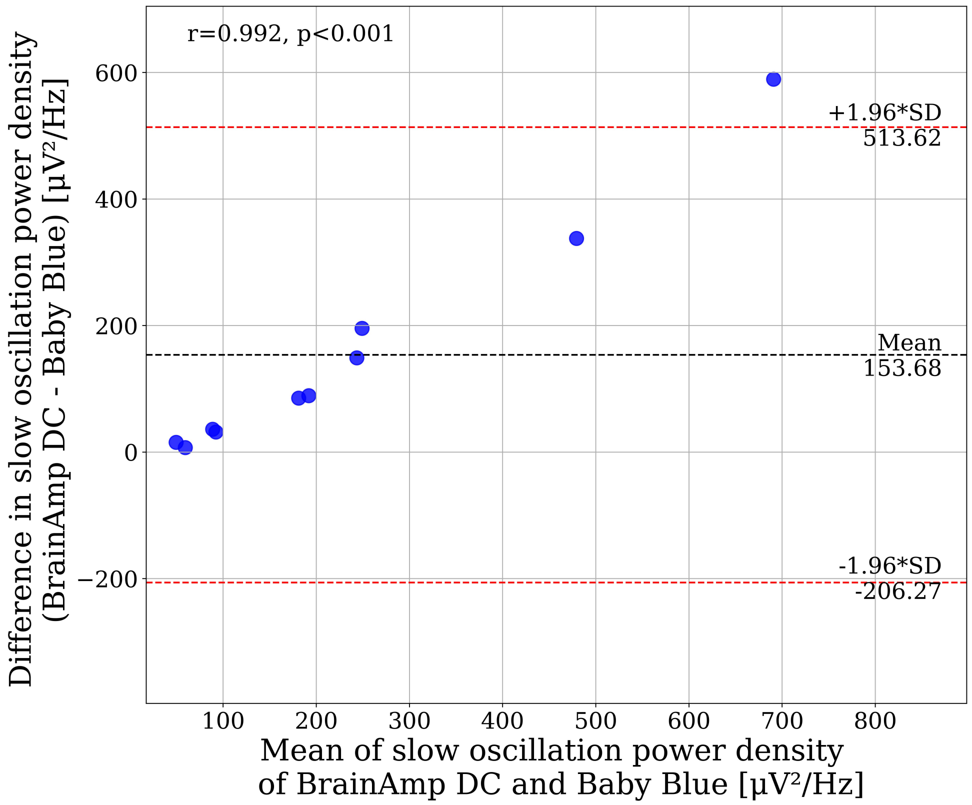

Figure 37: Bland–Altman plots of EEG power NREM sleep recorded at Cz. Mean EEG Slow Oscillation power from the BrainAmp DC and Baby Blue (y-axis) plotted against the difference in mean power (y-axis). Bandpass filter (0.48Hz - 53.32Hz) was applied on BrainAmp DC signals.

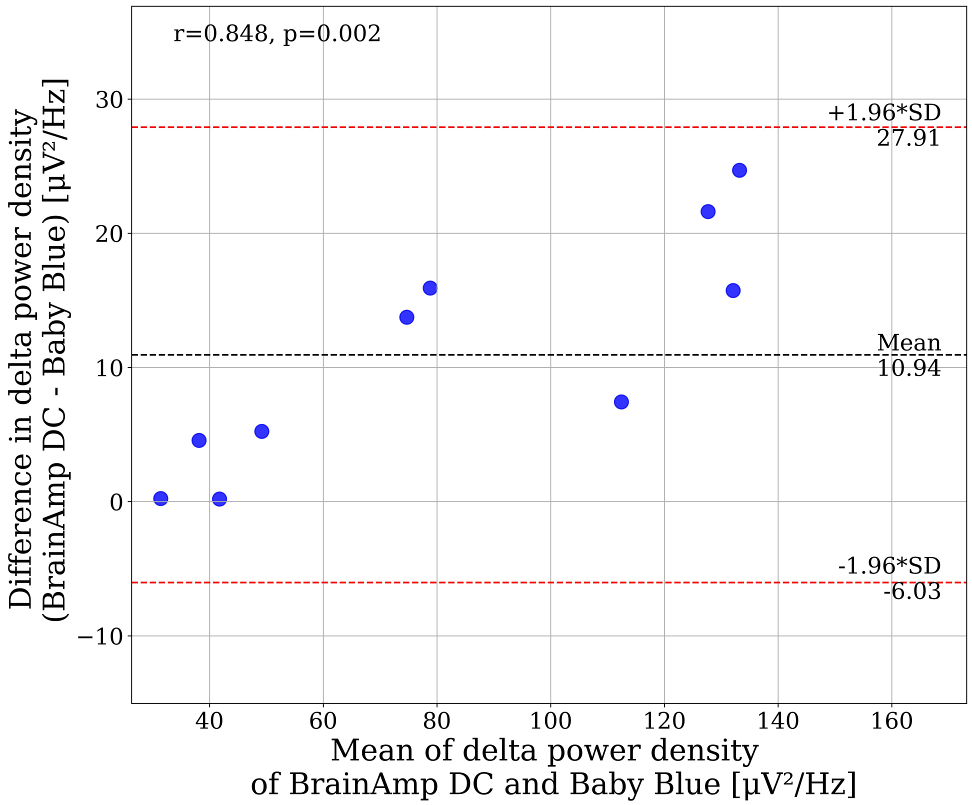

Figure 38: Bland–Altman plots of EEG power NREM sleep recorded at Cz. Mean EEG Delta power from the BrainAmp DC and Baby Blue (y-axis) plotted against the difference in mean power (y-axis). Bandpass filter (0.48Hz - 53.32Hz) was applied on BrainAmp DC signals.

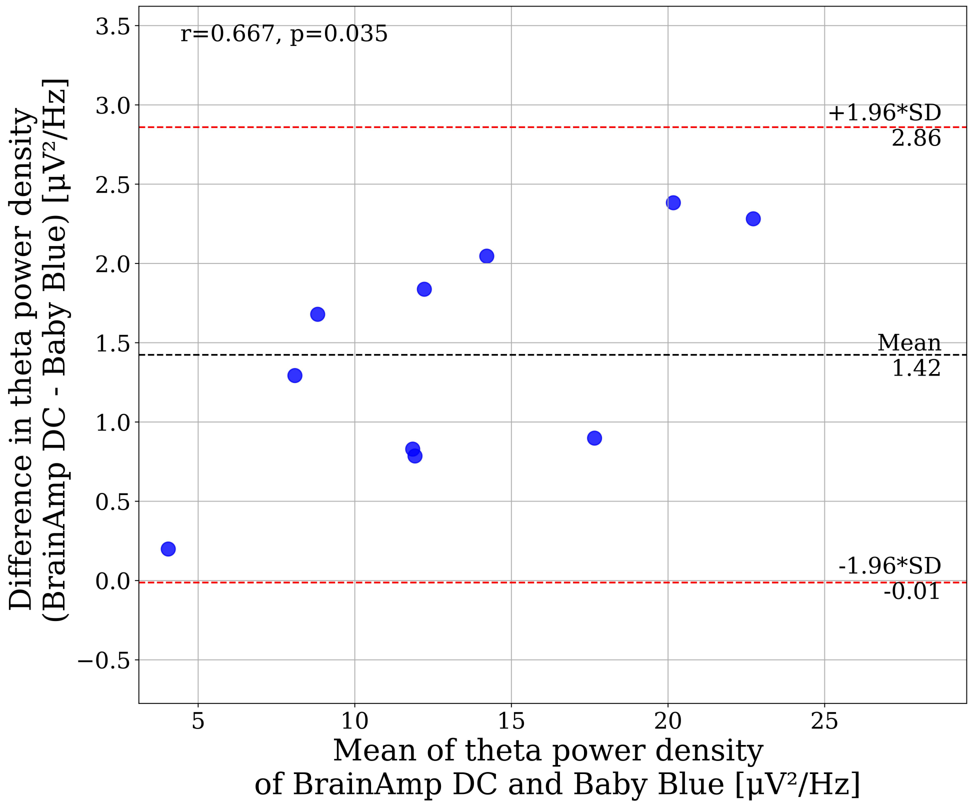

Figure 39: Bland–Altman plots of EEG power NREM sleep recorded at Cz. Mean EEG Theta power from the BrainAmp DC and Baby Blue (y-axis) plotted against the difference in mean power (y-axis). Bandpass filter (0.48Hz - 53.32Hz) was applied on BrainAmp DC signals.
